# Community based integrated wound care: Results of a pilot formative research conducted in Benin and Côte d’Ivoire, West Africa

**DOI:** 10.1371/journal.pgph.0002889

**Published:** 2024-02-09

**Authors:** Anita Carolle Akpeedje Wadagni, Théodore Ange Kouakou Yao, Gabriel Diez, Flora Houndjrèbo Balle, Aboa Paul Koffi, Paulin Aoulou, Marie-Hélène Zahiri, Parfait Djossou, Yves Thierry Barogui, Henry Assé, Jean-Gabin Houezo, Ghislain Emmanuel Sopoh, Mark Nichter, Roch Christian Johnson

**Affiliations:** 1 National Buruli Ulcer and Leprosy Control Programs, Ministry of Health, Cotonou, Bénin; 2 National Buruli Ulcer Control Programs, Ministry of Health and Public Hygiene, Abidjan, Côte d’Ivoire; 3 ANESVAD Foundation, Bilbao, Spain; 4 National Leprosy Elimination Program, Ministry of Health and Public Hygiene, Abidjan, Côte d’Ivoire; 5 Centre Interfacultaire de Formation et de Recherche en Environnement pour le Développement Durable, Université d’Abomey-Calavi, Abomey-Calavi, Benin; 6 Regional Institute of Public Health Comlan Alfred Quenum of Ouidah, University of Abomey-Calavi, Abomey-Calavi, Bénin; 7 School of Anthropology, University of Arizona, Tucson, AZ, United States of America; University of Oxford, UNITED KINGDOM

## Abstract

Appropriate treatment of chronic wounds is priority in the management of Neglected Tropical Skin Diseases (NTSDs) and non-communicable diseases. We describe an integrated, community-based wound care pilot project carried out in Benin and Cote d’Ivoire that entailed both outreach education and evidence based wound care training for nurses staffing rural clinics. This research was carried out by a transdisciplinary research. Following the collection of baseline data on wound care at home and in clinics, an innovative pilot project was developed based on a critical assessment of baseline data in three parts: a pragmatic nurse training program; mass community cultural sensitive outreach programs and a mobile consultation. It came out from our investigation that several dangerous homecare and inappropriate wound treatment practices in clinics, gaps in knowledge about Neglected Tropical Skin Diseases (NTSDs), and little health staff communication with patients about appropriate wound care. Nurse training covered 11 modules including general principles of wound management and advice specific to endemic NTSDs. Nurse pre-post training knowledge scores increased substantially. Eight mass community outreach programs were conducted, followed by mobile clinics at which 850 people with skin conditions were screened. Three hundred and three (35.65%) of these people presented with wounds of which 64% were simple, 20% moderate, and 16% severe cases. Patients were followed for ten weeks to assess adherence with wound hygiene messages presented in outreach programs and repeated by nurses during screening. Over 90% of simple and moderate cases were managed appropriately at home and 98% of wounds were healed. Of the 47 cases referred to the health center, 87% came for and adhered to wound care advice. In 90% of cases, wounds healed. This pilot study provides a model for introducing integrated community based wound care in Africa.

## Introduction

As a broad class of physical disorders, wounds and skin disease comprise one of the biggest burdens for health care systems worldwide. The Global Burden of Disease project ranked skin diseases as the fourth leading burden of nonfatal disease world-wide. In 2013, it was estimated that skin and subcutaneous diseases were responsible for 41.6 million disability-adjusted life years (DALYs) and 39.0 million years lived with disability (YLDs) [1]. Unfortunately, funding for research and community wound and skin care outreach has not been in step with the magnitude and gravity of this class of public health problems [2].

Wounds are the cause of considerable human suffering, disability, and stigma, all of which threaten household survival for those living on the margin [3]. Given their immense public health importance, the treatment of wounds has recently been recognized as a crosscutting issue in the control of NTSDs [4, 5]. For example, in studies of leprosy and lymphatic filariasis, wound self-care has been identified as an important and recurrent problem [6–10].

In the case of Buruli ulcer, the recent implementation of oral antibiotic therapy has increased the ability of these patients to be managed at home [11], thus necessitating home wound care. Similarly for diabetes, it is essential to care for wounds at home and recognize danger signs [12]. Assessment of the foot and skin at home is important to reduce serious complications requiring hospital care (amputation).

The potential role of community based integrated wound care programs as a means to identify NTSDs has already been demonstrated in the modest pilot projects in West Africa. For example, in Bankim, Cameroon, 328 cases of yaws were identified at mass Buruli ulcer (BU) outreach programs followed by screening camps over a three year period of time [13]. Prior to the outreach program, cases of yaws had not been seen at local clinics for years and the disease was thought to be eradicated. In Ouinhi, Benin, a proof-of-concept study on wound care integration was carried out in 2012. Chronic wound care was integrated into a community based decentralized BU program along with mass outreach programs. In addition to several cases of chronic wounds, many early stage (category I or II) BU cases were identified. Moreover, people who had refused centralized hospital treatment for BU accepted outpatient treatment at a local clinic. Appropriate care of the wounds, all of which healed well, had a powerful effect in demonstrating the effectiveness of this form of management. As a result, the status of both the clinic and nursing staff treating wounds increased [14].

Building on the experience of pilot programs such as these, a next logical step was to investigate how to develop well-coordinated community-based wound care outreach programs with two basic components. The first component is the retraining of nurses in best wound hygiene practices fitting the African context. The dual emphasis of such training programs are a pragmatic approach to best practices and better staff–patient communication in order to use wound treatment as a teachable moment [15]. The second necessary component is a culturally sensitive outreach program placing emphasis on home management of common wounds and recognition of the signs of NTSDs, infected wounds, and skin lesions. Core messages and practices promoted in the clinic and in community outreach must be consistent.

In this paper, we provide an overview of a community based integrated wound care project developed, pretested, piloted, and evaluated in Benin and Côte d’Ivoire, West Africa.

## Materials and methods

### Study setting

This project was conducted in two sites: the health districts of Divo in Côte d’Ivoire and Ouinhi in Benin.

The district of Divo is located 210 km from Abidjan, the capital city of Côte d’Ivoire. Covering an area of 3590 square kilometers, the district has a population of 404,821. Agriculture and petty trade are the main economic activities. The Divo Health District is co-endemic for several NTSDs such as BU, leprosy, and yaws. The health system in the district of Divo is consists of a network of 46 health centers and a regional hospital.

The district of Ouinhi is located in the Zou Department in southern Benin. Covering an area of 483 km^2^, Zou has an estimated population of 46,305. Like Divo, agriculture and petty trade are the main economic activities in the region and BU, leprosy, and yaws are endemic NTSDs. The health system of Ouinhi consists of four health centers and the *Centre Sanitaire et Nutritionnel Gbemoten* (CSNG) which serves as a reference center for the treatment of BU and other serious chronic wounds in Zou.

In addition to clinics associated with the biomedical health system, several other healing modalities and sources of medication are found in both Benin and Ivory Coast. These include drug peddlers in local markets, small pharmacies, traditional wound specialists who specialize in plant-based topical treatments and physiotherapy; and healers, diviners, and religious leaders who engage in the metaphysical treatment of wounds.

### Ethical and administrative aspects

The study was approved by the National Committee for Ethics in Health Research of Benin n° 43 of November 23th 2017 (N° 05/MS/DC/SGM/DRFMT/CNERS/SA). Technical notes about the study were sent to the health authorities in Côte d’Ivoire (N°72/MSHP/DC-PNLUB/HA/kaf) before the study was initiated. The local health authorities (district chief medical officer, health region coordinator) were informed about and provided guidance during the research project.

Informed consent was obtained orally from all adult participants in interviews and focus groups, and from parents, caretakers, or legal representatives of participants aged ≤18 years. Verbal consent was necessitated given both high rates of illiteracy and the need to provide details about the study in local languages. Most participants spoke only local languages. Written informed consent was obtained from all clinic staff interviewed.

Data were processed and analyzed with strict respect for confidentiality and anonymity.

### Conduct of study

A pre-post-intervention study on home-based and clinic-based wound care practices was carried out by a transdisciplinary team composed of clinicians, public health practitioners, a microbiologist, and social scientists. The study was carried out from 2017 to 2018 precisely in July 2017 in Côte d’Ivoire and from February to December 2018 in Benin. It was conducted in five stages:

1. Baseline data collection on wound care in the community and in local clinics staffed by nurses and nurse assistants;
2. Development and pre-testing of outreach education tools, clinic staff training modules, and wound management monitoring and evaluation tools;
3. Implementation of community and clinic-based wound care interventions, consisting of community outreach, mobile clinics, and clinic staff training workshops;
4. Evaluation of the effectiveness of outreach programs through assessment of improvement in basic wound care knowledge and practices;
5. Evaluation of clinic staff’s modification of wound management and communication with patients during screening camps and follow-up treatment.

The study was carried out in rural communities of both countries served by nurses and nurse assistants staffing local clinics. During the initial formative research stage, social scientist team members interviewed a wide variety of community stakeholders, including women having considerable experience treating wounds at home, community members presently or recently suffering from wounds and skin diseases (patients), traditional healers, and shop keepers selling allopathic and herbal medicines in the market. Team members with expertise in wound care interviewed and observed nurses in their clinics.

#### Primary outcome

Proportion of wounds managed appropriately at home after exposure to outreach programs and by nurses at clinics following wound care training programs.

#### Secondary outcomes

Development of practical and culturally appropriate training materials; shifts in wound care practices among nurses following training; number of participants attending community outreach programs; public understanding of key outreach program messages; number of patients visiting mobile clinics; proportion of patients with serious wounds referred to the health center; proportion of these wounds healed at the health center.

### Outreach program development

Baseline data collection was carried out by social scientists as a first step toward the development of a culturally sensitive outreach program. Social scientists from both countries participated in baseline data collection in each other’s country to enhance south-south exchange and team building. Five methods were used as a means of identifying home care practices in the community: open ended key informant interviews, semi-structured interviews, case studies, focus groups using visual prompts and what if scenarios (what would you do if …), and structured observations (**S1 Appendix**).

Findings from this formative research were used in the development of a wound care outreach program that combined the dissemination of basic information on wound hygiene principles with do’s and don’ts based on a critical review of common wound care practices in the community by clinician members of the research team.

An image rich modular program was designed to be delivered by PowerPoint projection to mass audiences. It was pretested in seven communities. A teach back method [16–18] was used when pretesting modules under development. During outreach programs, questions were posed to the community about core messages being conveyed and community members were asked to rephrase what they had heard in their own words. The outreach program was designed to be interactive. Social scientists took notes on community response messages both well and poorly received, questions raised, and doubts expressed. This data was used to revise the outreach program and prepare facilitators for questions and confrontations.

A modified outreach program was then piloted in nine communities in the two countries by social scientists working in concert with local clinicians. The outreach programs were followed by wound screening at mobile clinics the next day. Improvement in wound care practices were then documented during three rounds of follow up interviews and structured observations with community members who presented wounds and skin conditions at mobile clinics.

### Nurse training program development

The clinical training component of the study was implemented in three steps: baseline data collection, participation in a highly interactive training workshop, and post training evaluation of improvement in knowledge about wound care and NTSDs, observed wound care practice, and practitioner: patient communication about wound care at home. Details were provided in **S2** and **S3 Appendices**.

Nurses in training were encouraged to ask patients about their homecare practices during clinic consultations and to see wound treatment as a teachable moment to reinforce do’s and don’ts presented in community outreach programs. Nurses were trained to routinely ask the following four core questions:

- Did you use any product to clean the wound at home?
- Have you applied any product externally on the wound as treatment?
- Have you taken any medicine orally for wound care or to reduce pain?
- What challenges will you face in managing the wound at home based on your work and daily tasks?

In addition to didactic training, skill-based training was provided in the clinic. Wound dressing best practices were demonstrated and attendees had the opportunity to ask questions about each stage of wound cleaning and bandaging. Evaluation of actual improvement in wound care practice were made during mobile camps.

### Mobile clinics

Following outreach programs (described in section 3.2), mobile clinics were held and community members were invited to have wounds and skin conditions screened by nurses. Wounds were classified into three groups based on severity and whether the case could be managed at home or required treatment at the clinic or referral to the hospital. **Table 1** lists the criteria for wound classification. Observations were made of how nurses assessed, triaged, and treated wounds. As well as messages communicated to patients about wound care management at home. Attention was also paid to whether the messages delivered reinforced the basic wound hygiene principles presented in outreach programs.

**Table 1:** Criteria for wound classification.

Classification	Criteria
**Simple cases (to manage at home)**	• Wounds of small size (diameter <5cm) • Superficial wounds • Absence of hemorrhage • Clean red wound does not ooze, • No clinical signs of infection (fever, swelling, bad odor, widening of the wound) • Patient with good general condition (no fatigue and weight loss
**Moderate cases (to manage at home)**	• Not too big wound, large diameter between 5cm and 10cm • No clinical signs of infection • Patient in good general condition
**Severe cases (to be referred to health center / hospital)**	• Deep wound • Wide wound (GD> 10cm) • Wound with danger sign • Wound flows pus or bleeds • Wound localized on the face or joint • Patient with poor general condition • Suspected case of BU, leprosy, lymphatic filariasis

Eighteen months after the training, all nurse participants were interviewed by phone and asked whether the training they received proved viable in their clinics, and to comment on the challenges they faced when trying to implement best practices.

**Fig 1:**
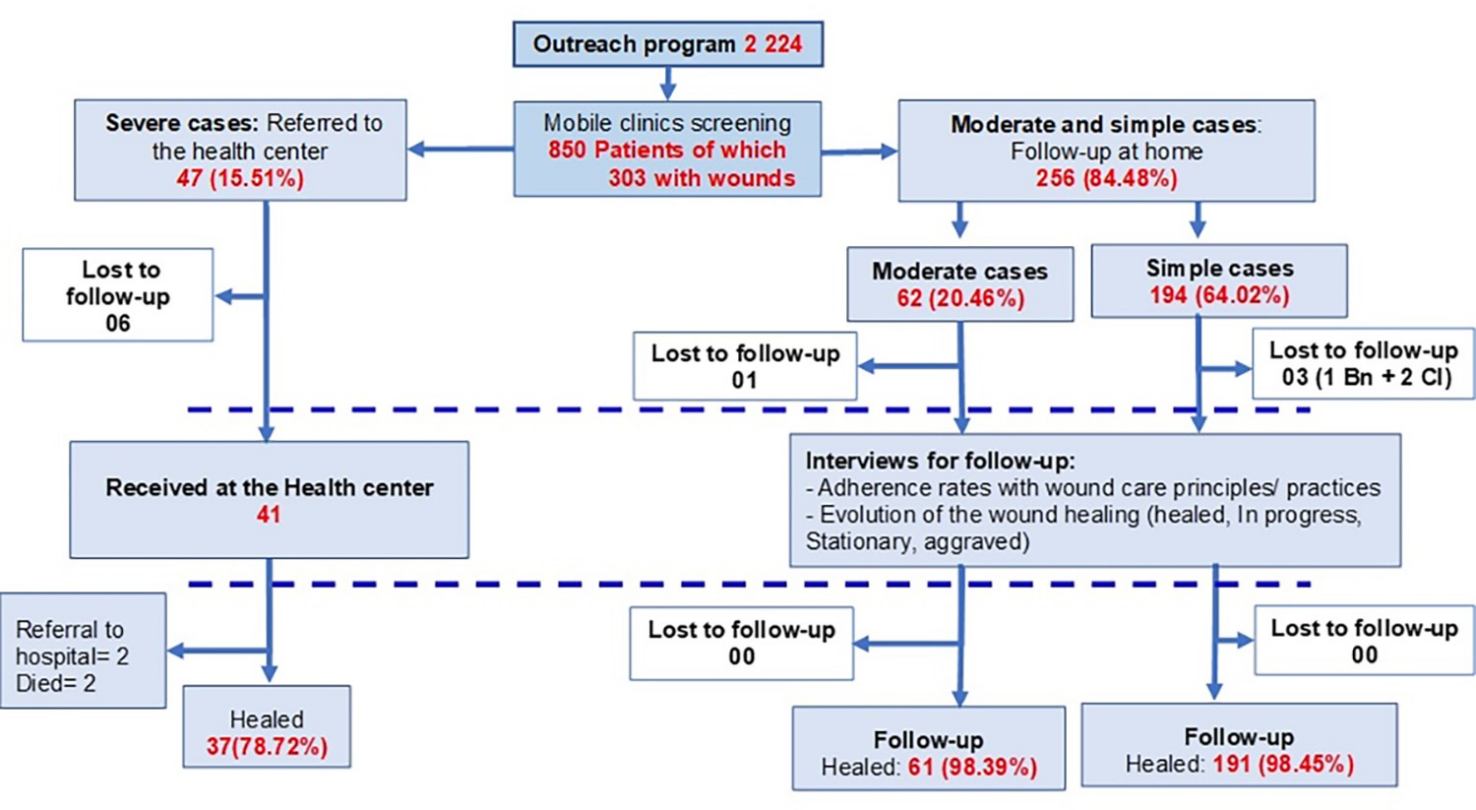
Diagram of the organization of activities and results of the wound care project.

**Fig 1** describes the model developed for evaluation of the appropriateness of wound care practices for three categories of patients assessed during screening. All cases were followed for ten weeks and visited three times. Assessment of home care for simple and moderate cases was carried out by a team of clinic health staff (doctors, nurses, and nurse-assistants), social scientists, and local community health workers.

### Data analysis

#### Outreach program

Baseline data on home based wound care practices was assessed by a panel of clinicians who determined whether they were negative, neutral, or positive. Following the outreach program, home care practices were monitored to see if negative practices declined, and basic wound hygiene practices were adopted.

#### Nurse training

An observation instrument previously developed to assess nurses’ wound care practices at a Buruli ulcer reference center in Benin was adapted and use in this project (**S4 Appendix**). Wound care behaviors described and observed at baseline and following training were scored as falling into one of three categories: full adherence, partial adherence, and poor adherence. To assess the effectiveness of training, both the content and process of training was queried in semi-structured, open-ended interviews. Pre and post-test levels of wound care knowledge were entered into Excel, analyzed by SPSS and trend curves constructed to compare shifts in knowledge and practice knowledge.

#### Shifts in home-based care

Data collected from follow up visits to community members who attended mobile clinics was analyzed using descriptive statistics that assessed core features of appropriate wound care management for each of the three groups of subjects classified by severity of their wound. The proportion of healed patients was calculated.

## Results

Results are presented for all three components of the project: community outreach, nurse training, and shifts in community based wound management.

### Community outreach

At baseline, 92 key informant and semi-structured interviews and 10 focus groups (10 participants per group) were conducted to assess existing wound care practices.

Several harmful wound care practices were identified in both countries, such as the use of scalding hot water on wounds, placing sand or ash on wounds to keep off flies, and the use of herbal medicines and the topical application of powder from antibiotic capsules to dry wounds. Two major challenges identified were the common perception in both countries that drying a wound was necessary for wound healing, and that it was beneficial to drain the pus from ulcers and abscesses by punctures or medicines that hasten their eruption. The products commonly used to treat wounds at home and in clinics identified in both countries during the baseline assessment, classified by frequency of use, effects and comments on the reasons for their use, are given in **Table 2**.

**Table 2:** Products commonly used to treat wounds at home and at clinic identified in the two countries during the baseline assessment, classified according to the frequency of use, their effect and comments on the raisons for using.

Common wound care practices and products	Negative	Positive	Country	Comments–reason for using
**Hot water on wound**	Very Common		Both countries	Reduce pain Clean wound
**Releasing pus water from wound**	Common		Both countries	
**Washing soap**		Less common	Benin	Clean the wound
**Plant *Ocimum gratissimum***		Common	Benin	Clean the wound
**Hot water**	Very common		Both countries	Clean wound
**Sand/Ash**	Less common		Both countries	Protect against flies
**Lemon**	Common		Both countries	Stop the bleeding
**Toothpaste**	Very common		Both countries	Dry the burn wound
**Antibiotic capsules-used externally**	Very common		Both countries	Dry the wound
**Ibuprofen**	Very common		Both countries	Reduce pain
**Herbal powder, bark and roots**	Common		both countries	Dry the wound
**Black soap prepared from plants**	Less common		Benin	Dry the wound
**(Agatouman/ sekoutoure (*chromolaena odorata)***		Very common	Both countries	Stop the bleeding
**Banana (*musa*) *sap***	Less common		Both countries	Treatment
**Hysope (*Hyssopus officinalis*)**	Less common		Both countries	Treatment
**Kaolin**	Common		Côte d’Ivoire	Dry the wound
**Red oil**	Common		Benin	Treatment
**Shea butter**		Less common	Both countries	Scar management
**Snail shell**	Less common		Both countries	Dry the burn wound
**Petrol and brake oil**	Less common		Both countries	Treatment

* Less common: < 50%; Common: 50–70%; Very common > 70%

Another challenge identified was a negative attitude about bandaging wounds. Bandaging a wound was thought to both call unwanted attention to one’s state of vulnerability, inviting witchcraft, and because there was widespread belief that exposure to air was necessary for wound healing (**Table 3**). At the same time, however, there was concern about flies being attracted to open wounds infecting them with small worms (*wevi*—Fon) leading to infection.

**Table 3:** Key misperceptions about wound management that needed to be included in outreach education and addressed during clinical training.

Misperceptions	Rationale
**Dry the wound**	Drying the wound speeds up healing
**Using of antibiotic**	Antibiotic reduces pain and scarring quickly
**No use of the bandage**	Bandage attracts the wrong look Bandage inhibits healing also causes stigmatization

A further challenge was the belief that oral consumption of antibiotic capsules was an effective remedy for pain associated with wounds. Positive wound management practices were also identified, such as the use of plants *Chromolaena odorata* to stop bleeding, and *Ocimum gratissimum* for cleaning wounds when soap was not available. Inclusion of these herbs in outreach programs was seen by the community as acknowledging local wisdom and well received (**Table 2**).

Notably, wound care practices in both countries were quite similar allowing for common messages to be developed and translated into local languages. At the center of the program was the formulation of do’s and don’ts messages based on an assessment of data found in **Table 3**. These messages are embedded in the outreach program available for review at the flowing website https://www.pnllub.org/wound-care/.

The outreach program was designed and delivered as a modular PowerPoint presentation. The presentation was pretested in seven mass outreach programs conducted in the two countries and attended by approximately 1550 participants. Based on feedback, the presentation was further modified and piloted in an additional nine communities attended by over 2100 community members. **Table 4** presents the summary of the numbers interviews and observations conducted per country and mass outreach event implemented per countries.

**Table 4:** Summary of the types and numbers of data collection methods and mass outreach event implemented per countries.

Method	Benin	Côte d’Ivoire
**Baseline** (participants)		
**Individual interviews**	(41)	(51)
**Focus in community**	(04)	(06)
**Observation**	(15)	(19)
**Outreach:** events (participants)		
**Pretest outreach**	03 (991)	04 (558)
**Pilot outreach**	04 (1246)	05 (856)

The evaluation of the outreach programs was extremely positive with the vast majority of informants stating that the programs were at once informative and understandable. Most gratifying, wound hygiene messages were among the three most commonly recalled messages by informants when asked what three messages they remembered from the outreach program. The three most commonly recalled messages were:

- The wound should be washed with potable drinking water and mild soap or tchiayo (*Ocimum gratissimum*) tea;
- No longer put powder of antibiotic capsules or other products on a wound, only shea butter;
- Wounds should no longer be left open; a bandage of clean cloth should be applied.

Interviews with women several weeks following attendance at outreach programs documented high knowledge retention related to wound hygiene, as well as common queries. The two most common queries were why some people’s wounds heal more quickly than others, and about the use of herbal plants (like tchiayo, *Ocimum gratissimum*) to reduced symptoms like itching.

Women were also asked if they had shared information about wound care with neighbors who had not attended outreach programs. Following their own positive experience of wound healing, several women reported that they now felt confident to reach out offer advice. The two most common messages passed along were: 1) “do not put anything on the wound” and 2) “clean the wound with potable drinking water and mild soap; apply shea butter and bandage.” The message “do not place antibiotics on the wound,” a common popular practice, was not commonly shared. Resistance to core outreach messages by a small minority of community members in both countries was also documented.

Notably, resistance was more commonly expressed by men than women. Men were also more inclined to support antibiotic use as a cheap and effective way of treating wounds, and reducing pain, applying a hot machete to wounds along with red oil, and to suspect witchcraft as a reason wounds did not heal.

### Training program

A total of 18 health clinic staff (twelve males and six females) were trained. Ten health clinic staff were trained in Cote d’Ivoire and eight in Benin. Among the trainees from Benin, four were assistant nurses with less formal medical training than nurses training.

Interviews conducted at baseline revealed considerable variability in knowledge about common NTSDs. There was greater knowledge about Buruli ulcer (90%) and traumatic wounds (80%) than yaws (10%), leprosy (40%), fungal lesions (60%), and scabies (60%). Structured observations of wound dressing likewise revealed considerable variation in wound dressing practices. Individual protection measures such as wearing a lab coat and gloves while dressing wounds were often not followed nor deemed important. The liberal use of unnecessary wound cleaning products such as polyvidone iodine was routine practice and nurses had no idea that these antiseptic products might actually impede the healing process by disturbing the skin microbiome.

Proper procedures for bandaging were commonly not followed. Bandaging principles not adhered to include applying the bandage centripetally (start from the end towards the center), and in case of hand or foot bandaging, not placing fine compresses between the fingers/toes to avoid creating maceration of the skin at interdigital spaces. The positioning of the patient during bandaging to prevent disabilities was often incorrect. Bandages placed near joints were not always applied in a functional position, and as a result did not allow articular movements, leading to articular stiffness.

Also notable was a general lack of communication between nurses and patients during wound dressing. Patients were not typically informed about the progress of wound healing in relation to the evolution of the healing process. While nurses were aware of harmful home treatments for wounds, they were not observed to communicate to patients why these practices were harmful. In short, they saw their role as providing treatment to patients, not education.

Interviews also revealed that there were no clear criteria for referral of chronic wounds or skin conditions to reference hospitals other than cases of category III BU and traumatic lesions requiring surgery. Nurses stated that once a patient was referred, they rarely received feedback about the patient from the reference hospital (**S5 Appendix**).

Some of the key points emphasized in each of the 11 modules is developed (**Table 5**).

**Table 5:** Title and contents of the training modules.

Module #	Title	Contents
1	Wound management and basic wound hygiene principles	Characteristics of the different layers of the skin Theoretical bases of wound management Different stages of healing Realization of appropriate dressing for each stage of healing.
2	Wound bandage	Objectives and principles of the bandage Techniques for bandaging wounds.
3	Buruli ulcer	Diagnosis Sampling techniques for biological confirmation Signs of gravity Management of a BU patient
4	Traumatic wounds	Definition of traumatic wounds Etiologies and management Severity factors Reference criteria
5	Burns	Recognizing signs of burn Severity cotation Treatment Management of complicated burns Classification and reference criteria.
6	Leprosy	Diagnosis of leprosy Treatment Prevention of complications.
7	Yaws	Diagnosis of yaws Biological confirmation Treatment and preventive measures.
8	Scabies	Diagnosis and treatment
9	Fungal lesions	Description of the types of fungal lesion Clinical diagnosis Treatment and prevention
10	Lymphatic Filariasis	Diagnosis and treatment
11	Cutaneous leishmaniasis	Diagnosis Treatment and prevention

The training was designed to be dynamic and interactive and was well received by participants. Teach back was found to be an effective way to make sure participants understood information presented [17]. Different participants were selected to provide a summary of the main points contained in each module in their own words. Clinical cases were volunteered by both teaching staff and nurse participants as a means of illustrating points raised and to ground discussion. Teaching analogies volunteered by teaching staff were greatly appreciated as a means of enhancing comprehension of complex topics, and to address patient’s queries. For example, nurses spoke of being confronted by patients who were disappointed when they did not receive expected medications. These patients could not understand why medicines were being freely administered to other patients (such as Buruli ulcer patients) with wounds perceived to be similar to their own. The clinician trainer shared an analogy he used in such situations. Sometimes two people look like twins, but they are not twins and have very different personalities, likes, and dislikes. Like that, wounds may look alike on the surface, but be quite different and require different treatment. This is the reason nurses sometimes perform tests to determine what kind of wound a patient is experiencing.

In some instances, the feasibility of wound care practices promoted in the workshop were vigorously debated. For example, nurses were taught that the systematic use of antiseptic (gentian violet, polyvidone dermic) was unnecessary for wound cleaning. Some nurses stated that patients expected its use and worried that patients might question the quality of care they were receiving if antiseptic was not used. In short, they drew attention to the symbolic importance of the practice and they encouraged people to assess the clinical signs of infection to decide on the use of specific antiseptics. Discussion ensued about how a nurse could reassure patients. It was pointed out that non-use of products on wounds was a core message being promoted in outreach programs. Continuity of core messages was recognized by all nurses as necessary.

Nurse participants evaluated both the content and process of the training workshop. The feedback received was unanimously positive and significant improvement in knowledge were documented in areas where there had been major gaps at baseline (**S5 Appendix**).

The time allocated for the training was judged sufficient and the content of modules pertinent to the practices of nurses staffing local clinics. Participants particularly valued the interactive process of teaching and the chance to discuss practical considerations of wound care provision. Nurse assistants found some of the information in the modules beyond their level of comprehension given their level of education. They appreciated the teach back method as this allowed them to hear information expressed in local language, by analogy, and through case presentation.

Post training assessment at 18 months revealed that nearly all nurses were willing and able to implement what they had learned during training. Only one nurse assistant continued to use “gentian violet” on superficial wounds despite being trained to do otherwise.

With respect to challenges, nurses reported that some patients were initially skeptical about the use of soapy water to clean wounds and non-use of polyvidone dermic. However, after explanations were provided to them, they accepted the new practice. The same held true for patients who directly requested prescriptions for anti-inflammatory medications and antibiotics. Explanations as to why these were unnecessary was generally accepted. A key lesson learned by nurses was that nurse: patient communication was vital to treatment adherence. Taking time to explain actions to patients made a huge difference.

### The community intervention

**Fig 2:**
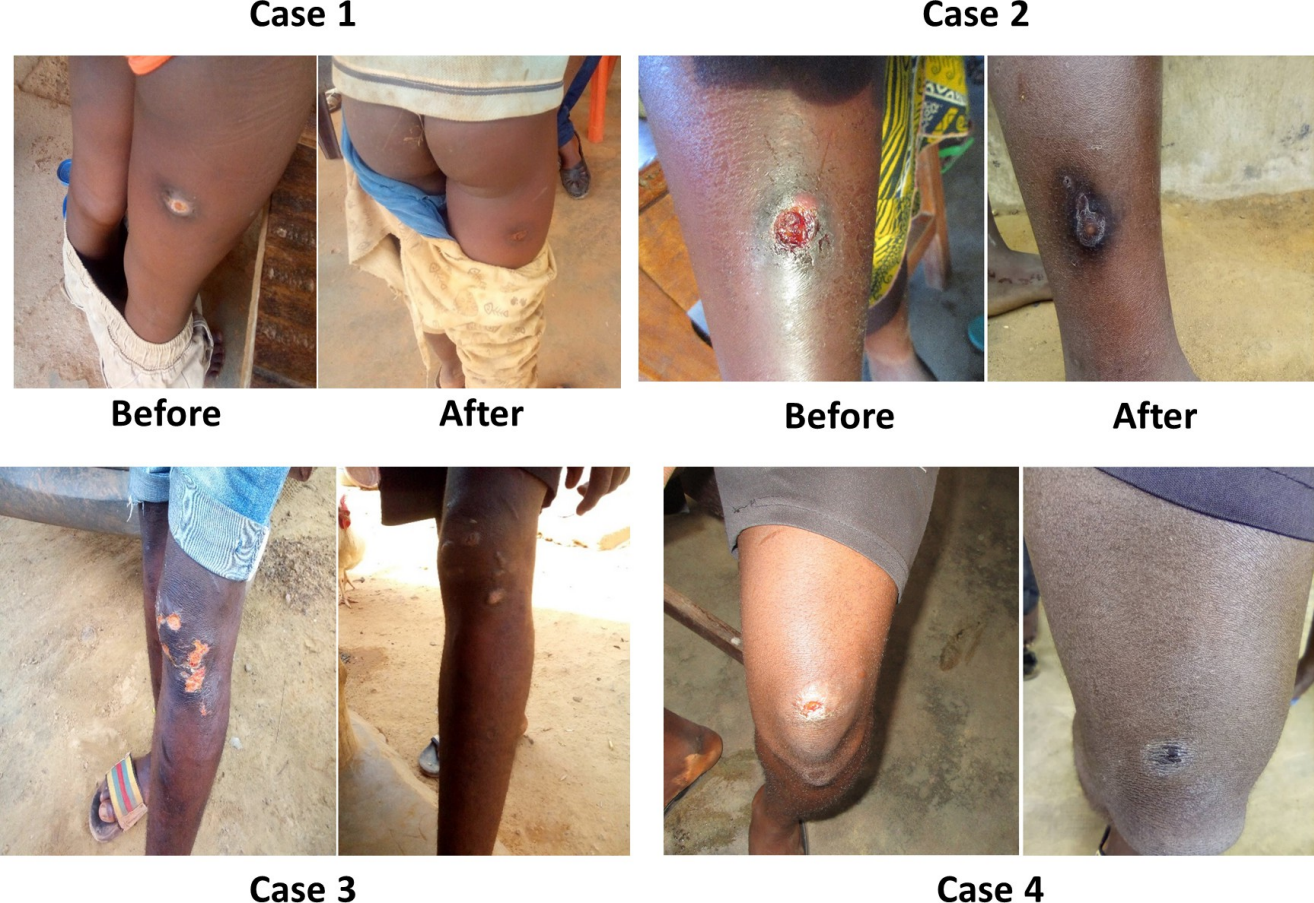
Clinical images.

Eight hundred fifty people with wounds and skin conditions were examined during eight mobile clinics following outreach events. Of the 850, 303 (35.65%) were suffering from wounds of varying levels of severity. **Table 6** presents data on the demographics of cases and the number of wounds seen by category of severity. A majority of patients with wounds, (N = 226; 74.59%) were younger than 15 years and male 64.7% (N = 106), The vast majority (84%) of wound cases were manageable at home **(Fig 2)**. Only 47 of the 303 wound cases were referred to hospital for treatment (**Table 6**).

**Table 6:** Types of diseases and classification (NTSDs and others).

	Benin n (%)	Côte d’Ivoire n (%)	Total n (%)
**Wounds (n = 303)**			
Buruli ulcer*	3 (2.33)	0 (0.00)	3 (0.99)
Acute/traumatic wound	88 (68.22)	104 (59.77)	192 (63.37)
Infected wound	6 (4.65)	64 (36.78)	70 (23.10)
Chronic ulcer	32 (24.81)	6 (3.45)	38 (12.54)
** Total**	**129 (100)**	**174(100)**	**303(100.00)**
**Classification of wound (n = 303)**			
Simple cases	84 (65.12)	110 (63.22)	194 (64.03)
Moderate cases	28 (21.71)	34 (19.54)	62 (20.46)
Severe cases	17 (13.18)	30 (17.24)	47 (15.51)
**Total**	**129 (100.00)**	**174 (100.00)**	**303 (100.00)**
**Gender**			
Male	**76 (58.91)**	**120 (68.97)**	**196 (64.7)**
Female	**53 (41.09)**	**54 (31.03)**	**107 (35.30)**
**Total**	**129 (100.00)**	**174 (100.00)**	**303 (100.00)**
**Age (years)**			
<15	**95 (73.64)**	**131 (75.29)**	**226 (74.60)**
≥ 15	**34 (26.36)**	**43 (24.71)**	**77 (25.40)**
**Total**	**129 (100.00)**	**174 (100.00)**	**303 (100.00)**
**Others skins conditions (n = 547)**			
Tinea capitis	115 (26.62)	61(53.04)	176 (32.18)
Scar	115 (26.62)	0 (0.00)	115 (21.02)
Other cutaneous mycoses	94 (21.76)	29 (25.22)	123 (22.49)
Bacterial dermatoses	36 (8.33)	0 (0.00)	36 (6.58)
Eczema/Urticaria	50 (11.57)	6 (5.22)	56 (10.24)
Scabies	7 (1.62)	19 (16.52)	26 (4.75)
Abscess	3 (0.69)	0 (0.00)	3 (0.55)
Lichen plan	6 (1.39)	0 (0.00)	6 (1.10)
Psoriasis	6 (1.39)	0 (0.00)	6 (1.10)
**Total**	**432 (100.00)**	**115 (100.00)**	**547 (100.00)**
**Grand total**	**561 (100.00)**	**289 (100.00)**	**850 (100.00)**

*Confirmed by PCR

Most community members who attended the mobile clinic suffered from skin diseases (N = 547). These cases included scabies, *Tinea capitis*, and cutaneous mycoses (Table 6). Patients presenting with these skin conditions received appropriate care according to their condition. It was beyond the scope of the project, however, to follow them up: they were referred to the nearest hospital for the follow up. **Table 7** summarizes treatment provided for skin conditions seen at the mobile clinic.

**Table 7:** Summary of treatment and advice provided for three common skin diseases diagnosed during at mobile clinics.

Diagnostic	Treatment provided		Advice
	The day of mobile mass clinics	At home	
**Tinea capitis**	• Griseofulvin tablet or syrup • Ketoconazole cream	• Griseofulvin tablet or syrup • Ketoconazole cream • Duration: at least 06 weeks	• Wash regularly with soap and water • Wear clean clothes
**Eczema**	Antihistaminic tablet or syrup	Antihistaminic tablet or syrup	Referral to the health center for follow-up
**Scabies**	1st application of scabicidal solution	2nd application of the product 24 hours after the first application	• Wash clothing and bedding in hot, soapy water • Avoid applying the product on the face • Avoid contact of the product with children • Treatment at home of all contacts subjects

Three rounds of interviews were conducted with community members who presented with wounds at the mobile clinic at weeks 2, 6, and 10. The study retention and treatment adherence rate were very high. Of the 256 people suffering from simple and moderate wounds followed at home, 252 (98.43%) remained in the study. Eighty-two percent of these patients properly cleaned their wounds with potable drinking water and mild soap and 205 (80.7%) followed advice not to apply any product to their wounds during the healing process, including antibiotic powder. Eighty-eight percent of the 41 people who did not clean their wounds appropriately were male, 58.8% of the 51 people who placed inappropriate products in wounds were female. Appropriate bandaging of wounds was undertaken by 139 (54%) patients (43.8% female, 56.2% male). Shea butter application was undertaken by 76% of females and 24% males. All mild to moderate cases healed by the time of the third interview.

Of the 47 cases referred to the health center, 41 patients (87.23%) reported to the clinic for treatment. Of the six patients lost to follow up, two moved out of the study area, one judged the distance between home and the health center to be too far, and three from Côte d’Ivoire changed their residence and could not be located. The wounds of 37 (90.24%) healed following treatment and the other four patients were still under treatment at the last follow up. A notable improvement was observed in the appearance of the lesions between screening and the end of the follow-up period.

Changes in communication between nurses and patients were assessed during screening and during follow-up clinic observations. At baseline, communication between nurses and patients was minimal and primarily directed toward compliance with treatment offered. Patients were not told anything about their wound or how healing was progressing. Following training, nurses embraced the treatment of wounds as a teachable moment for educating patients about wound hygiene, and an opportunity to review treatment do’s and don’ts based on responses to queries about a patient’s wound care behavior before attending the clinic.

A process evaluation found that a sustained working relationship was established between the hospital, clinic staff, and community health workers. Continuing education was also documented as taking place. Feedback on patients triaged to the hospital was provided to nurses in the referring clinic. Nurses learned through experience which referrals were warranted and how to manage cases when released from the hospital. Nurses provided feedback to both community health workers and patients about the quality of their home care and the evolution of their wounds. An impact evaluation revealed that each stakeholder in the health care system felt more comfortable contacting other members for health issues beyond wound care and NTSDs.

## Discussion

The primary objective of this wound care pilot project was to develop a model for delivering community outreach and nurse training suitable for the West African context. This project was designed and implemented by a transdisciplinary team of clinicians, public health specialists, microbiologists, and social scientists. The pilot project consisted of five stages: a) baseline data collection on wound care management in the community and in local clinics staffed by nurses and nurses’ assistants; b) development and pre-testing of both outreach education modules and clinic staff training modules; c) development of wound management monitoring and evaluation tools; d) implementation of interventions, consisting of clinic staff trainings, mass community outreach events, and mobile clinics; and e) patient follow-up as a means of assessing improvement in wound care behavior at home and in the clinic by those attending screening camps.

Important lessons were learned during each stage of outreach related research. During the baseline collection phase, we learned that general questions about wound care did not evoke good responses from community members. Adding visual prompts and asking about concrete situations proved far more productive. We also learned that presenting “what if” scenarios and the collection of case studies provided important insights into gender roles in wound care and health care decision making. This topic will be the subject of a separate publication.

During pretesting of the outreach program, we learned not to present too much information to the audience at one time. It was far more prudent to scale back on information about many kinds of wounds and to focus on a smaller set of conditions with a central message: wound hygiene is necessary for the healing of all wounds. During pretesting we identified common challenges to basic wound hygiene messages posed by community members. Two of the biggest challenges were related to inappropriate antibiotic use: “do not put antibiotics from capsules purchased in the market on wounds” and do not consume antibiotic capsules to control pain. Convincing responses to these challenges had to be developed.

We also learned that the use of culturally appropriate teaching analogies was an effective way to introduce new ideas about wound care by tying new ideas to existing knowledge about cooking, agriculture, etc. However, teaching by analogy had to be done with care as we learned during pretesting that not all teaching analogies proposed by the research team members proved to be effective (**S6 Appendix**).

Another lesson learned was that in the outreach programs it was better to use images of wounds that are not very severe as these images evoke fear and are a distraction. It was better to present images of common wounds and skin diseases and early to moderate cases of NTSDs.

In the piloting phase of outreach related research, we learned that teach back was an effective method of instruction in large groups. After a few core messages were introduced, the audience was asked questions and invited to voice their understanding of key messages presented in their own words. Community members enjoyed the exercise and were quite vocal.

The seven main findings of the project were:

1. A majority of common wounds and skin conditions in rural Africa diseases can be managed at home with minimal cost if appropriate wound hygiene principles are followed. In our pilot study, 84% of the wounds presented by people who came for screening at a mobile clinic could be successfully managed at home.
2. Mass outreach programs are popular in the community and attract a wide variety of people suffering from chronic wounds and skin diseases [13, 14, 19, 20]. Mass outreach events focusing on wound management in our study acted as a magnet for cases of NTSDs and skin diseases. In our pilot study, three cases of BU and 26 cases of scabies were identified.
3. Implementing mass outreach events in rural areas increases the status of community health workers and trust in nursing staff. And it creates closer working relations between community health workers, nursing staff in rural clinics and doctors working in reference hospitals.
4. Nurses are willing to change their wound treatment practices if they participate in respectful and interactive training programs such as that described in this paper. A major challenge identified at the beginning of this project was whether clinic staff wedded to longstanding and somewhat antiquated wound care procedures, and a public accustomed to receiving medications from nurses for wounds would accept advice largely focused on basic wound hygiene [21]. We found that when nurses had the opportunity to share experiences, weigh the pros and cons of recommended practices, and consider the pragmatics of implementing best care practices in their local context they were willing to change their behavior and assess the results.
5. Wound care behavior change is also possible in the community. When the public is informed about the do’s and don’ts of wound care in outreach programs, they are willing to try the strategy and see what happens. The demonstration effect of following good wound hygiene principles reinforces outreach messages, and leads to dissemination of information in the community.
6. The biggest challenge to fully accepting wound care best practice advice in the community involves antibiotic use for self-treatment. The topical and oral misuse of antibiotics for wound self-treatment has been reported to be common in many Low or Middle Income Countries (LMICs). Misuse of antibiotics impedes healing by disturbing the skin microbiome and may be a factor fostering antibiotic resistance. In our project sites in Benin and Cote d’Ivoire, resistance to giving up antibiotics for pain related to wounds and as a wound care quick fix appears to be more common among men. Messages tailored to men will need to be developed in the future.
7. A final finding is that teaching nurses and community health workers simple communication skills increases their confidence in providing information about wound care and responding to questions and challenges posed by community members. Previous studies in Benin and Cameroon [22–24] found that poor communication between hospital staff and patients compromises quality of care. In this project, attention was focused on effective ways of communicating with uneducated community members in a culturally appropriate manner. This proved beneficial both in outreach programs and in the clinic where nurses were faced with a need to explain to patients why they were not cleaning wounds with betadine (antiseptic) and providing antibiotics. Teaching by analogy [23, 25] was one method found to be effective in both outreach and nurse training programs, as was the use of evocative pictures and scenarios.

To date, little research has been conducted on wound care practices in LMICs and development of culturally appropriate approaches to wound care outreach. Our approach to wound care training and outreach is unique for several reasons. First, our approach is based on ongoing formative research conducted by a transdisciplinary team participating in all stages of research. Social scientists inform clinicians about common home care practices and clinicians assess the potential efficacy and harm of these practices. This is a fundamental step in developing a culturally sensitive do’s and don’ts approach to wound care outreach education. Once clinicians are made aware of wound care misconceptions, they work in concert with social scientists to identify the best ways to address misconceptions in a culturally compelling manner. In our study clinicians came to appreciate why it was necessary to take the time to address wound healing misconceptions and inform patients why some common home care practices actually impede wound healing.

A second unique feature of our approach is a commitment to blend best practices and best pragmatics given the exigencies of the local context and the need for a low-cost approach to wound care. In our study, an inventory was initially made of locally available inexpensive materials for cleaning and dressing wounds. Whenever possible, use of effective natural products like shea butter was encouraged.

Third, non-confrontational and culturally appropriate communication skills are a core competency and essential part of outreach facilitation and clinician training. This included practical training in how to address common challenges during both mass outreach events and during clinical encounters.

Fourth, feedback loops are established to insure sustained communication and continuing education. In our study, nurses who referred cases to the hospital were informed if the referral was appropriate or if the patient could be managed locally at the clinic. Nurses likewise provided information to community health workers about cases referred and patients were provided with feedback on home treatment and the progression of wound healing. Particular attention was paid to teaching community members about the danger signs of wound infection.

Four additional contributions of the project may briefly be highlighted. First, an observational tool (https://www.pnllub.org/wound-care/) was developed to assess the effectiveness of wound management in the clinic pre and post interventions. Second, 11 nurse training modules were developed and normed for use in West Africa, and an interactive process of implementation elaborated (https://www.pnllub.org/wound-care/). Third, a process for developing community-based outreach messages was developed. The image-rich PowerPoint-driven outreach program is found on our website (https://www.pnllub.org/wound-care/). A future publication will describe in detail the process of developing, pretesting, and implementing mass outreach events. Fourth, a model for evaluating the effectiveness of outreach and clinical training was developed. This model enables an assessment of improvement in wound care behavior in both the community and clinic for wounds of different degrees of severity.

We would argue that wound care and the management of common skin diseases is best conceived as a crosscutting issue that needs to be integrated into primary care, NTSDs, sexually transmitted infection, water, sanitation, and hygiene (WASH) [26], and school health programs. In the case of NTSDs, we would further argue that focusing more attention on general wound care in the community will act as a magnet for the early detection and treatment of diseases like BU, yaws, and scabies. Community outreach enabling the early detection and treatment of neglected tropical skin diseases and chronic skin conditions is also cost effective. It reduces the economic burden for both households and the health care system given the high cost of treating advanced cases of NTSDs and chronic wounds in hospitals. The need to reduce this economic burden has been emphasized in the 2030 agenda for sustainable development [27].

### Limitations of the study

We make note of three study limitations. The first limitation relates to the pre–post evaluation of wound care at home by patients attending screening camps. We did not question patients we followed after attending the mobile clinic about their previous home care practices. We assumed their previous home care practices reflected community wide wound care patterns already documented as problematic in most cases. A second limitation of the study is that content of the modules used in trainings were designed for qualified nurses.

In Benin, in Ouinhi district, some clinic staff were less educated nurses’ assistants. In the future, separate training materials will need to be developed for this group. Third, in this community-based intervention we focused far more on treating wounds than common skin diseases, although medication was provided to those afflicted. In the case of scabies, the community was informed about how to recognize this disease during outreach programs, but it was beyond the scope of the project to follow up scabies and other skin disorder cases.

## Conclusions

This study demonstrates that mass wound care outreach programs followed up by mobile clinics are an effective way of identifying both acute and chronic wounds in need of management as well as NTSDs. The study also found that, with a little basic wound care education, the majority of wounds identified during outreach screenings can be successfully managed at home following simple wound hygiene principles. When introduced in a culturally sensitive manner, community members, especially women, are ready to adopt new, low to no cost wound care practices. The study further found that nurses who attend training workshops like the one described in this paper are willing to change their wound management procedures and adopt best local practices for wound care in Africa.

Wound care is a cross-cutting public health priority for NTDs, non-communicable diseases, and primary care programs in LMICs. We would argue that wound care management be integrated in these programs as well as a broad-based WASH strategy [26, 27] with its emphasis on water-washed diseases and sanitation. We further propose that the lessons learned and the methods employed in this wound care project have broad public health application for developing outreach programs and culturally sensitive clinical training courses for other health conditions.

## Supporting information

S1 Appendix Methods used during formative research. (DOCX)

S2 Appendix Nurse training module development. (DOCX)

S3 Appendix Literature review for training materials. (DOCX)

S4 Appendix Home care monitoring. (DOCX)

S5 Appendix Evaluation of shifts in nurse knowledge. (DOCX)

S6 Appendix Examples of teaching analogies. (DOCX)

S1 Text Example of an interview with healthcare personnel. (DOCX)

S2 Text Example of an interview with healthcare personnel. (DOCX)

S3 Text Example of an interview with healthcare personnel. (DOCX)

S1 Data Screening and follow up of patients Benin. (XLSX)

S2 Data Screening and follow up of patients Cote Ivoire. (XLSX)

S3 Data Benin and Cote Ivoire health agent. (XLSX)
