# Supplementary material for: Community based integrated wound care: Results of a pilot formative research conducted in Benin and Côte d’Ivoire, West Africa
Source: PLOS Glob Public Health. 2024 Feb 9;4(2):e0002889. doi: 10.1371/journal.pgph.0002889 (PMC10857723; doi:10.1371/journal.pgph.0002889)
Supplement: S1 Appendix — (DOCX) [file pgph.0002889.s001.docx]

**Methods used during formative research to identify local wound care perceptions and practices**

Key informant interviews were initially conducted with community members in both countries who a) currently or recently suffered from wounds or b) were identified as having considerable experience treating wounds. These interviews generated a list of common wound care practices and products used in home care of wounds. This data was used in semi structured follow-up interviews and focus groups carried out in community and clinic settings to further explore wound care perceptions and practices inclusive of:

- - Substances commonly used to clean, treat, and bandage wounds;
  - Perceptions of the wound healing process;
  - Substances used to treat wound related sensations (e.g., pain, itching, burning sensation);
  - Recognition of wound related danger signs.

During mobile clinic screening, social scientists were afforded another opportunity to collect additional data on home based wound care, and to ask those attending the mobile clinic key messages they recalled from the outreach program the day before.
