# Supplementary material for: Community based integrated wound care: Results of a pilot formative research conducted in Benin and Côte d’Ivoire, West Africa
Source: PLOS Glob Public Health. 2024 Feb 9;4(2):e0002889. doi: 10.1371/journal.pgph.0002889 (PMC10857723; doi:10.1371/journal.pgph.0002889)
Supplement: S2 Appendix — (DOCX) [file pgph.0002889.s002.docx]

**Nurse training module development**

A critical review of baseline data on nurse knowledge of NTSDs and wound care practices identified gaps in both that needed to be addressed in training modules.

The modules drew upon state-of-the-art wound care principles found in the literature (Appendix N°3) and were designed to foster discussion about what nurses deem feasible in the local context given resources at hand. Modules also presented do’s and don’ts based on the critical assessment of baseline data. While under development, the modules were presented to a panel of experts composed of clinicians, public health workers, and professors of medicine and public health from Benin and Cote d’Ivoire. The panel reviewed the modules and suggested changes in the content.

The modules were presented in highly interactive and dynamic workshops in both countries. Participants were asked to raise questions, express doubts, and share experiences with patients.
