# Supplementary material for: Community based integrated wound care: Results of a pilot formative research conducted in Benin and Côte d’Ivoire, West Africa
Source: PLOS Glob Public Health. 2024 Feb 9;4(2):e0002889. doi: 10.1371/journal.pgph.0002889 (PMC10857723; doi:10.1371/journal.pgph.0002889)
Supplement: S3 Appendix — (DOCX) [file pgph.0002889.s003.docx]

**Literature review for training materials development**

The ‘Teach back’ method originally designed to improve practitioner: patient communication was adopted and adapted by workshop instructors. Workshop participants were called upon to restate in their own words core messages in the training. Pre and post-tests documented knowledge change.
