# Supplementary material for: Community based integrated wound care: Results of a pilot formative research conducted in Benin and Côte d’Ivoire, West Africa
Source: PLOS Glob Public Health. 2024 Feb 9;4(2):e0002889. doi: 10.1371/journal.pgph.0002889 (PMC10857723; doi:10.1371/journal.pgph.0002889)
Supplement: S4 Appendix — (DOCX) [file pgph.0002889.s004.docx]

**Home care monitoring**

Changes in wound care practices was documented by social scientists who conducted structured observations in the homes of participants one to two weeks after screening at mobile clinics, at week 6, and then at week 10. An observation grid, designed by clinician members of the team, was used to monitor wound care practices. Practices documented included:

- - Wound hygiene: cleaning the wound, with potable drinking water and mild soap;
  - Dressing materials used: no application of decoctions, antibiotics, powder, etc., application of shea butter;
  - Bandaging: use of gauze or clean cloth; no use of leaves;
  - Evolution of the wound: healed, healing in progress, little change, wound has become infected, wound needs to be referred to health center.

Observations were also made about gender roles in wound care decision-making, and whether women who attended the outreach programs disseminated information in their community by offering advice to other women about wound care.
