# Supplementary material for: Community based integrated wound care: Results of a pilot formative research conducted in Benin and Côte d’Ivoire, West Africa
Source: PLOS Glob Public Health. 2024 Feb 9;4(2):e0002889. doi: 10.1371/journal.pgph.0002889 (PMC10857723; doi:10.1371/journal.pgph.0002889)
Supplement: S5 Appendix — (DOCX) [file pgph.0002889.s005.docx]

**Evaluation of shifts in nurse knowledge pf wound care best practices**

The three objectives of clinic staff training were to: 1) improve the level of wound care and NTSDs knowledge, 2) increase staff willingness to accept new wound care best practices after critical assessment of what is feasible in the African context, and 3) raise clinician awareness of local wound perceptions and practices leading to a more proactive approach to patient communication during wound treatment as a teachable moment. Trainings of five days duration were conducted in each country. The training was modular and problem based. Modules were developed after reviewing best practices gleaned from the International NTSDs and wound care literature and critical assessment of baseline data collected in the first stage of the project. Major gaps in knowledge, points of confusion, and do’s and don’ts related to both clinic and home-based wound care practices were emphasized in the training (<https://www.pnllub.org/wound-care/>). To assess success of nurse training, each Nurse (N) and nurse’s assistant (AN) asked a series of questions to be covered in each module before instruction and following instruction. Pre: post test results were compared. Shifts in wound care and communication practices were observed during mobile clinics.
