# Supplementary material for: Community based integrated wound care: Results of a pilot formative research conducted in Benin and Côte d’Ivoire, West Africa
Source: PLOS Glob Public Health. 2024 Feb 9;4(2):e0002889. doi: 10.1371/journal.pgph.0002889 (PMC10857723; doi:10.1371/journal.pgph.0002889)
Supplement: S6 Appendix — (DOCX) [file pgph.0002889.s006.docx]

**Examples of teaching analogies**

Teaching analogies proved an effective means of conceptual translation, but analogies had to be carefully pretested. An example of a successful teaching analogy involved asking people to think about weeds that grow in maize fields.

Just as weeds prevent maize from growing well by sapping moisture and nutrients from the soil, so placing products on wounds to dry them does not allow wounds to heal well as this requires a moist clean environment. An example of an analogy that did not prove effective, although it sounded viable to the team, was comparing wound healing to laying the foundation of a house.

The analogy proposed was that to lay a good house foundation brick made of soil need to be dried in the sun slowly to become strong. If the drying process is hastened the bricks are not strong and the foundation is weak. This was compared to treatments that attempt to dry wounds quickly resulting in weak wound foundation. In both cases, one ends up spending more money on repairs to a poorly built house or a poorly treated wound. While community members understood the analogy, it was not well remembered.
