# Supplementary material for: Community based integrated wound care: Results of a pilot formative research conducted in Benin and Côte d’Ivoire, West Africa
Source: PLOS Glob Public Health. 2024 Feb 9;4(2):e0002889. doi: 10.1371/journal.pgph.0002889 (PMC10857723; doi:10.1371/journal.pgph.0002889)
Supplement: S1 Text — (DOCX) [file pgph.0002889.s007.docx]

# **ENTRETIEN PERSONNEL DE SANTE**

NOM ET PRENOM :

Niveau : BEPC

Profession : Aide-soignant fonctionnaire

**SECTION A : INFORMATION SUR LE PERSONNEL DE SANTE**

1. Centre de santé :
2. Nom :
3. Age :
4. Sexe : Masculin
5. Ethnie :
6. Niveau le plus élevé de scolarisation :

2^ème^ cycle

1. Adresse

| Région | District | Aire sanitaire | Localité/village |
| --- | --- | --- | --- |
|  |  |  |  |

1. Profession:

Aide- soignant

**SECTION B : Connaissances et attitudes du personnel soignant**

B : Quelles sont les différents types de plaie que vous connaissez ?

A : Il y a des plaies dues aux infections qu’on incise. Il y a d’autres qui viennent avec des plaies très infectées, d’autres viennent avec des plaies dues aux coups de machette dont on fait la suture, on soigne et ça finit. On reçoit des plaies de l’Ulcère de Buruli. Si on n’arrive pas à les prendre en charge, on les réfère. Il y a les plaies très infectées, les abcès.

B : Recevez-vous en consultation des patients porteurs de plaie ? Si Oui, combien en moyenne par jours ? Mois ?

A : En fonction du mois, on peut recevoir vingt et cinq malades

B : Quelles sont les étiologies/causes des plaies que vous recevez ?

A : Si on prend la plaie due aux coups de machette, c’est que la personne s’est blessé à la machette. Ça c’est une cause. Si on prend les plaies très infectées, nous ne sommes pas sur le même terrain qu’eux, on ne sait pas comment ça s’est passé. Quand on leur demande, ils disent que c’est un « petit truc » qu’ils ont négligé, c’est pour cela que c’est devenu comme ça. Souvent on leur demande de venir à l’hôpital pour se faire soigner. Pour les plaies dues à l’ulcère de Buruli, on leur demande comment cela a débuté, quand ils nous indiquent, on fait les premiers pansements et on les évacue.

B : Quelles sont les différentes étapes par lesquelles passe une plaie avant de guérir selon vous ? Quels produits utilisez-vous couramment pour chaque étape ?

A : S’il y a une plaie qui est très infectée, la première étape on reçoit le malade, on l’enregistre, on fait la prescription  du dakin, des compresses et on fait des pansements quotidiens pendant une semaine. Une fois la plaie bien propre, on entame le pansement avec le savon et après la bétadine jaune.

B : Quels sont les produits de pansements à votre disposition ?

A : On a la bétadine rouge et la bétadine jaune. On ne dispose pas de Dakin. On le prescrit et le malade va lui-même l’acheter.

B : Quelle est la fréquence de changement des pansements ? Pourquoi ?

A : Il y a des pansements à deux jours et les pansements quotidiens. C’est-à dire que si la plaie est bien propre et due à une blessure à la machette, on finit la suture et on lui rendez-vous deux jours après pour le deuxième pansement.

Si c’est une personne qui est venue avec une plaie très infectée, on fait le pansement au dakin et on lui donne rendez-vous le lendemain. Ça dépend des types de plaies. Si c’est un abcès qu’on a incisé, on fait le pansement au dakin et le malade revient le lendemain pour le prochain pansement jusqu’ à ce que le pus s’arrête. On poursuit par la suite avec la bétadine jaune.

Quels sont selon vous les critères de changement des pansements ?

1. Comment évaluez-vous l’évolution d’une plaie que vous prenez en charge ?

B : Pensez-vous que les pansements que vous réalisez sont adaptés aux types de plaie ?

A : Je pense bien que c’est adapté parce que si quelqu’un s’est blessé à la machette et vient.

On fait le pansement pendant une à deux semaines, si la plaie finit, c’est que le pansement est adapté. Soit en cas d’abcès, si on commence le pansement avec le dakin et qu’on voit que la suppuration est arrêtée, c’est que le pansement est adapté. Si c’est une question d’UB, on ne fait rien. On lui fait un papier et on le réfère. On lui fait le premier pansement avant de le référer à Divo parce qu’on n’a pas les moyens nécessaires pour s’en occuper.

B : Prescrivez-vous des antibiotiques aux malades dans le cadre de la prise en charge de leurs plaies ? Si oui, quels antibiotiques prescrivez-vous ?

A : Pour les autres types de plaies, on prescrit les antibiotiques sauf le cas de l’ulcère de Buruli. A présent comme je ne suis pas le prescripteur, c’est l’infirmier qui est autorisé à répondre à cette question.

B : Prescrivez-vous des antalgiques aux malades dans le cadre de la prise en charge de leurs plaies ? Si oui, quels antalgiques prescrivez-vous ?

A : C’est du ressort de l’infirmier.

B : Est-ce que vous référez les malades porteurs de plaies vers d’autres hôpitaux ? Si oui, vers quels hôpitaux les référez-vous ?

A : Ici, si l’on veut référer un malade, comme nous sommes un centre de santé rural, on l’envoie au CHR de Divo

B : Est-ce que les hôpitaux de référence vous font une contre-référence ?

A : Je ne sais pas. Souvent nos parents viennent et nous dise qu’ils ont été bien reçus et que tout va mieux. Souvent, ils viennent avec les malades et quand on les voit, on est satisfait.

B : Souhaitez-vous être formé sur les soins des plaies ?

A : Cela dépend de vous. Si vous voulez nous former plus, parce que Chiépo est un gros centre où on reçoit beaucoup de malades. Voilà ce monsieur, c’est parce que l’annonce est passée qu’il est arrivé. Sinon c’est un gros centre et il y a beaucoup de plaie. Je veux être formé pour sauver la population de chiépo.

**SECTION C : Renforcement de capacité des agents de santé**

**B :** Avez-vous jamais reçu une formation sur la prise en charge des plaies ?

- - - Si oui, quand et où ?
    - nombre de jour de formation ?
    - thématique abordée ?
    - Appréciation de la formation

A : Oui à Vavoua pour l’UB. C’est en 2014. La formation a duré un jour. On a commencé à huit heures et on a fini à dix-sept heures trente. Elle a porté sur les plaies UB. La formation était bonne. J'étais même le représentant de l’UB à Vavoua.

B : Si non souhaiteriez-vous être formé sur la prise en charge des plaies ? Si oui sur quels aspects ou quelles thématiques ?

A : J’aimerais qu’on me forme encore pour avoir plus de connaissances. Je voudrais qu’on me forme sur tout jusqu’à comment soigner une plaie.

B : Avez-vous d’autres suggestions à faire pour l’amélioration de vos connaissances et compétences en matière de gestion de plaies ?

A : ce que je peux suggérer, puisque vous êtes les responsables de là-bas, on n’a pas une salle de pansement, une table de pansement ; c’est ce qui nous crée des problèmes.

Aussi, le matériel de pansement : il y a juste des ciseaux, pinces à disséquer, pince gourge.

Une bonne boite de petite chirurgie.

# **GUIDE D’ENTRETIEN PERSONNEL DE SANTE (COMMUNAUTE)**

Questionnaire No : ……………… Date de l’interview : ……/………/………

**SECTION A : INFORMATION SUR LE PERSONNEL DE SANTE**

1. Centre de santé : …………………………………………………
2. Nom : …………………………………………………………….

**SECTION B : Types de pratiques observées chez les patients et nature des questions posées par les patients**

9. Recevez-vous en consultation des patients porteurs de plaie ?

10. Quelles sont les étiologies ou causes des plaies que vous recevez ?

B : À quel stade de la plaie les malades viennent-ils vous voir ?

A : En tout cas s’il s’agit de plaies infectées, je ne sais pas comment qualifier le stade. Souvent, les plaies qui viennent sont à un stade avancé.

B : Ont-ils reçu un traitement à la maison avant de venir à vous ? Si oui, de quel traitement à domicile s’agit-il ?

A : Forcé. Mais on ne sait pas. Avant-hier, le patient qui est venu et qu’on a référé, il a dit qu’il mettait de la potasse dans la plaie. Certains disent qu’ils mettent de la pénicilline en poudre ; en cas d’abcès, les patients disent qu’ils ont fait ‘canaris’.

B : Quand les patients viennent, leur demandez-vous, les pratiques essayées à domicile avant de venir au centre de santé ?

A : Tout ce qu’ils mettent sur la plaie n’est pas bon chez moi. Tout est mauvais. Ils donnent du tétanos aux gens.

15. Dans l'affirmative, quelles sont les pratiques positives ou négatives les plus courantes en matière de soins des plaies, que ce soit au domicile ou ailleurs ?

Positives

Négatives

B : Les malades ont-ils l’habitude de nettoyer leurs plaies ? Si Oui, qu'utilisent-ils ?

A : Avant de mettre les produits là-dessus, je ne sais pas.

B : Les recouvrent-elles ou les bandent-elles ? Si Oui avec quoi ?

A : Oui avec des chiffons très sales

B : Est-ce que les malades posent des actes dangereux pour la santé ? Si oui quelles sont ces pratiques dangereuses ?

A : Oui les malades posent des actes dangereux. Si quelqu’un ramasse, quelqu’un a une plaie, imaginons la compresse que le petit a mis sur sa plaie, c’est une compresse vraiment souillée. Cette compresse porte les germes. Même le microbe du tétanos, ce n’est peut-être pas la plaie qui va le tuer mais plutôt le tétanos. On leur dit tout cela.

B : Prennent-ils des produits / médicaments dangereux pour la santé ? Si oui, quels sont ces produits / médicaments ?

A : Ils n’ont jamais fait venir ces produits ici. Quand ils viennent, on leur pose la question de savoir quel médicament ils ont mis sur la plaie. Seulement le vieux a dit qu’il a mis de la potasse sur sa plaie. Il est allé dans la rue pour acheter ses médicaments. On leur dit que les médicaments qu’ils achètent dans la rue sont toxiques pour leur santé. On leur demande de venir voir l’agent de santé qui va prescrire des médicaments et soigner leurs plaies.

Ils voient que la plaie évolue bien, ils sont contents.

B : Est-ce que les malades ont des interdits / restrictions alimentaires quand ils ont des plaies ? Si oui, quels sont ces interdits / restrictions alimentaires ?

A : A ma grande connaissance, si c’est une plaie diabétique, on donne des conseils, ce qu’il doit consommer ou pas. Pour les autres plaies, comme on ne fait pas la glycémie pour savoir si la personne est diabétique ou non. Donc sur les autres plaies, je ne peux pas donner une réponse exacte. Les malades peuvent tout manger sauf le diabétique.

B : Quelles sont les pratiques des malades pour le soin des cicatrices ?

A : Si on commence le pansement avec la bétadine jaune, si on voit que cela e ne s’améliore pas, on leur conseille de payer du banéocin pommade ou poudre. Comme il contient des antibiotiques, cela est plus rapide. Quand les malades payent, au bout d’une semaine, la plaie est finie.

B : Quelles sont les pratiques des malades pour la prise en charge de la douleur ?

A : Ce sont des comprimés qu’on doit prescrire. Ce sont les infirmiers qui le font.

B : Quelles sont les pratiques des malades pour la prévention des invalidités ?

A : On demande de faire une rééducation.

29. Est-ce qu’il y a des conceptions / idées fausses de la plaie et sa guérison chez les patients qui influencent leurs pratiques ou leurs recherches de traitement ? (ex : l’idée qu’il faut sécher la surface de la plaie)

B : Quels types de question les malades vous posent-ils sur leurs blessures (plaie) ?

A : Bon ! Quand ils viennent d’abord, c’est nous qui posons la première question. On leur demande comment la plaie a commencé.

B : Les malades suivent-ils généralement les conseils que vous leur donnez ou suivent-ils le traitement recommandé ?

A : Par voie orale ? Chaque fois on leur demande de faire venir les médicaments pour savoir s’ils les prennent correctement. On demande au malade de venir avec ses médicaments au deuxième rendez-vous. Ils suivent vraiment le traitement et les conseils.

B : Merci monsieur.
