# Supplementary material for: Community based integrated wound care: Results of a pilot formative research conducted in Benin and Côte d’Ivoire, West Africa
Source: PLOS Glob Public Health. 2024 Feb 9;4(2):e0002889. doi: 10.1371/journal.pgph.0002889 (PMC10857723; doi:10.1371/journal.pgph.0002889)
Supplement: S2 Text — (DOCX) [file pgph.0002889.s008.docx]

# **GUIDE D’ENTRETIEN PERSONNEL DE SANTE**

Questionnaire No : ……………… Date de l’interview : ……/………/………

**SECTION A : INFORMATION SUR LE PERSONNEL DE SANTE(CLINIQUE)**

1. Centre de santé : CSR de Sakota
2. Nom :
3. Age : 43 ans
4. Sexe : Masculin
5. Ethnie : Baoulé
6. Niveau le plus élevé de scolarisation :

Aucun niveau Primaire 1^er^ cycle 2^nd^ cycle Universitaire

1. Adresse

| Région | District | Aire sanitaire | Localité/village |
| --- | --- | --- | --- |
|  | Divo | Sakota | Sakota |

1. Profession:

Infirmier

**SECTION B : Connaissances et attitudes du personnel soignant**

B : Quelles sont les différents types de plaie que vous connaissez ?

A : il peut avoir une plaie hémorragique, il peut avoir la plaie propre et la plaie avec les éléments nécrosés dedans. Je ne sais pas si la notion de type donne allusion au mobile de la plaie ou quoi. Mais je crois qu’il y a la plaie hémorragique, la plaie non hémorragique mais propre, et la plaie sale.

B : Recevez-vous en consultation des patients porteurs de plaie ? Si Oui, combien en moyenne par jours ? mois ?

A : Oui on en reçoit. On a au moins six pansements par jour soit 100 ou 120 pansements par mois.

B : Quelles sont les étiologies/causes des plaies que vous recevez ?

A : On est en milieu et la plupart de nos plaies sont causées par des objets coupants notamment les machettes. Il peut avoir des plaies dues aux AVC, aux accidents de moto, il peut avoir aussi un abcès. Mais généralement les plaies qu’on a sont dues aux objets coupants.

B : Quelles sont les différentes étapes par lesquelles passe une plaie avant de guérir selon vous ?

A : Dans le cas d’une plaie hémorragique, il va falloir arrêter l’hémorragie, faire le pansement de sorte que la plaie devienne propre. La plaie en devenant propre évite de s’infecter pour permettre à la peau de se régénérer.

Dans le cas d’une plaie nécrosée, il faut faire un pansement pour décaper les tissus morts, rendre la plaie propre, permettre à la peau de régénérer.

B : Quels produits utilisez-vous couramment pour chaque étape ?

A : Dans le cas de la plaie hémorragique, souvent le pansement simple avec les antiseptiques notamment la bétadine et autre et puis un pansement avec une compresse, un pansement bien fait. Si c’est une plaie nécrosée, on va utiliser le dakin pour décaper les tissus morts jusqu’à ce que la plaie devienne propre et cela permet à la peau de se régénérer. Les antiseptiques que sont le dakin et la bétadine sont les produits utilisés pour les pansements.

B : Quels sont les produits de pansements à votre disposition ?

A : Il y a la bétadine jaune et le dakin. Gants et compresses.

B : Quelle est la fréquence de changement des pansements ? Pourquoi ?

A : Cela dépend du type de plaie. Si c’est une plaie opératoire, on peut faire les pansements à quatre jours ; si c’est une plaie opératoire avec un drain notamment dans les hydrocèles, on fera le pansement de façon journalière. Si c’est une plaie nécrosée, une plaie qui suppure, on ne fera pas quatre jours. Les pansements dépendent du type de plaie. Pour une plaie nécrotique, on peut faire le pansement tous les deux jours avec le dakin. Pour une plaie qui suppure on fait un méchage et le pansement de façon quotidienne. Au fur et à mesure que la plaie devient propre, la fréquence des pansements baisse.

B : Quels sont selon vous les critères de changement des pansements ?

A : Ce sont l’évolution et le type de plaie.

B : Comment évaluez-vous l’évolution d’une plaie que vous prenez en charge ?

A : plusieurs critères d’évaluation de l’évolution d’une plaie : l’essentiel dans un pansement est d’assurer le confort du malade dans un premier temps et puis limiter le risque d’infection. Donc je reçois quelqu’un qui est blessé, qui est en train de saigner ; si tu ne fais rien le blessé se vide de son sang et son pronostic vital est en jeu. Si j’ai pu faire des points de suture pour arrêter l’hémorragie, j’ai évolué de façon favorable. Si je lui fais un pansement et qu’il dit qu’il a dormi et que la plaie n’a pas suppuré après un ou deux jours, la plaie évolue bien.

Dans le cas d’un abcès chaud et douloureux qui empêche le malade de dormir, et que j’ai incisé et que le malade est soulagé et qu’il y a moins de pus sur la mèche, je me dis que la plaie évolue bien. Puisque je tends vers la cicatrisation qui est l’objectif. Il faut aussi veiller à ce que la cicatrice ne limite pas les mouvements du membre.

B : Pensez-vous que les pansements que vous réalisez sont adaptés aux types de plaie ?

A : A parti du moment où on prend les gens qu’on soigne et que la plaie guérit, c’est que les pansements sont adaptés jusqu’à ce que de nouvelles normes viennent nous dire qu’on peut faire mieux. L’objectif est de lutter contre l’infection, guérir la plaie du malade afin qu’il reprenne son activité.

B : Prescrivez-vous des antibiotiques aux malades dans le cadre de la prise en charge de leurs plaies ? Si oui, quels antibiotiques prescrivez-vous ?

A : Oui c’est systématique. Pour certaines plaies simples, on peut donner l’amoxicilline simplement, le flagyl ou l’amoxicilline + acide clavulanique. On dit qu’on lutte contre l’infection parce qu’il y a une porte d’entrée, tu es donc obligé de faire une couverture antibiotique.

B : Prescrivez-vous des antalgiques aux malades dans le cadre de la prise en charge de leurs plaies ? Si oui, quels antalgiques prescrivez-vous ?

A : Oui puisqu’on a parlé de confort du malade ; s’il a mal, il faut lutter contre la douleur. On utilise donc les antalgiques. On peut prescrire du paracétamol pur, dans certains cas un anti-inflammatoire mais avec une bonne couverture antibiotique

B : Est-ce que vous référez les malades porteurs de plaies vers d’autres hôpitaux ? Si oui, vers quels hôpitaux les référez-vous ?

A : Oui beaucoup. Souvent on a des plaies qui durent10, 11 années, quand tu mets en place ton protocole de traitement et que la plaie ne guérit pas, c’est qu’il y a quelque part une bactérie. J’ai donc référé ces malades au district sanitaires où l’on a découvert après des examens une tuberculose. On a donc référé beaucoup de malades porteurs de plaies dans d’autres services. Maintenant toutes les plaies sont référées au pavillon UB du CHR de Divo.

B : Est-ce que les hôpitaux de référence vous font une contre-référence ?

A : Bon le service UB nous fait une contre-référence. Souvent c’est oralement et non par écrit. Souvent le malade revient avec son document de traitement qui fait office de contre-référence. Dans les autres services, ils ne nous font pas de contre-référence.

B : Souhaitez-vous être formé sur les soins des plaies ?

A :

**SECTION C : Renforcement de capacité des agents de santé**

B : Avez-vous jamais reçu une formation sur la prise en charge des plaies ?

- - - Si oui, quand et où ?
    - nombre de jour de formation ?
    - thématique abordée ?
    - Appréciation de la formation

A : De façon spécifique, non. En dehors de ce qu’on a appris à l’école, on n’a plus eu de formation sur la prise en charge des plaies.

B : Si non souhaiteriez-vous être formé sur la prise en charge des plaies ?

Oui non

Si oui sur quels aspects ou quelles thématiques ?

Si non pourquoi ?

A : Oui parce que c’est toujours bon d’apprendre. Il faut identifier les types de plaies et leurs origines dans un premier temps et leurs étiologies. Le programme national de lutte conte l’ulcère de Buruli nous a formé les éléments diagnostiques de la prise en charge de l’ulcère de Buruli. On a eu également une formation en dermatologie au pavillon de l’UB. On souhaite avoir ce genre de formation de façon régulière.

# **GUIDE D’ENTRETIEN PERSONNEL DE SANTE (COMMUNAUTE)**

Questionnaire No : ……………… Date de l’interview : ……/………/………

**SECTION A : INFORMATION SUR LE PERSONNEL DE SANTE**

1. Centre de santé : Sakota
2. Nom :

**SECTION B : Types de pratiques observées chez les patients et nature des questions posées par les patients**

B : Recevez-vous en consultation des patients porteurs de plaie ?

A : Oui

B : Quelles sont les étiologies ou causes des plaies que vous recevez ?

A : traumatiques, infectieuses

B : À quel stade de la plaie les malades viennent-ils vous voir ?

A : A tous les stades. Le malade se coupe le bras ou le pied après être allé au champ, il se retrouve à l’hôpital avec une plaie qui saigne. Aussi des cas de plaie avec les décoctions là-dessus, des feuilles noires jusqu’à ce qu’ils soient fatigués.

B : Ont-ils reçu un traitement à la maison avant de venir à vous ? Si oui, de quel traitement à domicile s’agit-il ?

A : Généralement parce que au champ, le patient écrase des feuilles qu’il applique sur la plaie avant de venir à l’hôpital. Ces feuilles sont appelées « sékou touré » ils la mettent dans la plaie qui saigne car il arrête l’hémorragie. Ils font par la suite un bandage.

B : Quand les patients viennent, leur demandez-vous, les pratiques essayées à domicile avant de venir au centre de santé ?

A : quand les patients ne saignent pas, ils ne viennent pas systématiquement à l’hôpital. Ils font de l’automédication avec des poudres noires qu’ils appliquent sur la plaie. Ils ne viennent à l’hôpital que lorsque la plaie saigne. On ne connaît pas la composition de cette poudre noire.

B : Dans l'affirmative, quelles sont les pratiques positives ou négatives les plus courantes en matière de soins des plaies, que ce soit au domicile ou ailleurs ? Positive ? Négatives ?

A : On pense que ces pratiques ne sont pas positives. Le temps qu’ils mettent pour soigner la plaie aurait été plus bénéfique s’ils venaient à l’hôpital. Et puis la cicatrisation de la plaie va durer ; ils s’exposent à d’autres maladies en dehors de la plaie telle que le tétanos. La poudre qu’ils écrasent se fait à même le sol ou sur une pierre en contact avec la poussière. La plaie risque aussi de s’infecter et évoluer vers la suppuration voire la septicémie. Le traitement devient donc plus cher. Je pense qu’il n’est pas efficace de faire la prise en charge au village. Quels que soient le niveau et la grandeur de la plaie, elle se traite facilement à l’hôpital.

B : Les malades ont-ils l’habitude de nettoyer leurs plaies ? Si Oui, qu'utilisent-ils ?

A : Ils chauffent l’eau, on utilise un chiffon pour frotter la plaie. Une fois nettoyée, ils appliquent la poudre sur la plaie.

B : Les recouvrent-elles ou les bandent-elles ? Si Oui avec quoi ?

A : Souvent, ce sont des morceaux de pagne qu’ils utilisent pour le bandage. Souvent, le morceau de pagne pose problème. Ils utilisent des morceaux de pagne sales.

B : Est-ce que les malades posent des actes dangereux pour la santé ? Si oui quelles sont ces pratiques dangereuses ?

A : La gestion même de la plaie les expose notamment à des infections et à une grosse maladie appelée le tétanos. Si le tétanos se trouve dans la terre et tu utilises un tissu souillé, tu t’expose au tétanos et aux bactéries qui sont dangereux pour la santé

B : Prennent-ils des produits / médicaments dangereux pour la santé ? Si oui, quels sont ces produits / médicaments ?

A : Pour ceux qui veulent utiliser des médicaments autres que les plantes, ils achètent les médicaments au marché et les appliquent sur les plaies. Généralement, on parle de « toupaye », de « téao », il y a aussi des pommades à la pénicilline, de la poudre, même du charbon noir

B : Est-ce que les malades ont des interdits / restrictions alimentaires quand ils ont des plaies ? Si oui, quels sont ces interdits / restrictions alimentaires ?

A : je n’ai jamais entendu parler d’interdits dans la communauté.

B : Quelles sont les pratiques des malades pour le soin des cicatrices ?

A : Je ne pense pas qu’il y ait des soins particuliers. Je ne connais pas une pratique rurale après une cicatrisation.

B : Quelles sont les pratiques des malades pour la prise en charge de la douleur ?

A : Il y a certaines racines ou tiges qu’on écrase et qui lutte contre la douleur. Ce ne sont pas des choses que je connais mais j’en ai entendu parler. Tu écrases, tu bois et cela calme la douleur.

B : Quelles sont les pratiques des malades pour la prévention des invalidités ?

A : Ils ne savent pas prendre en charge les invalidités.

B : Est-ce qu’il y a des conceptions / idées fausses de la plaie et sa guérison chez les patients qui influencent leurs pratiques ou leurs recherches de traitement ? (ex : l’idée qu’il faut sécher la surface de la plaie)

A : Oui il y en a en milieu rural. Notamment dans le cas de la lèpre où tu es considéré comme maudit. Même il y a beaucoup de spéculations sur l’ulcère de Buruli. Il y a beaucoup d’idées qui ne sont pas réelles concernant les plaies. Pour l’UB, on parle de plaie mystérieuse.

B : Quels types de question les malades vous posent-ils sur leurs blessures (plaie) ? Est-ce qu’ils ont des préoccupations particulières concernant leurs plaies ou leurs traitements ?

A : Le patient est généralement inquiet quand il arrive à l’hôpital. Mais c’est à nous de les rassurer que lorsqu’ils suivent correctement le traitement, on va arriver à les guérir.

B : Les malades suivent-ils généralement les conseils que vous leur donnez ou suivent-ils le traitement recommandé ?

A : Oui beaucoup le font.

B : Merci.
