# Supplementary material for: Community based integrated wound care: Results of a pilot formative research conducted in Benin and Côte d’Ivoire, West Africa
Source: PLOS Glob Public Health. 2024 Feb 9;4(2):e0002889. doi: 10.1371/journal.pgph.0002889 (PMC10857723; doi:10.1371/journal.pgph.0002889)
Supplement: S3 Text — (DOCX) [file pgph.0002889.s009.docx]

# **ENTRETIEN PERSONNEL DE SANTE**

**A= ENQUETE**

**B= ADMINISTRATEUR**

Nom :

Age : 53 ans

Ethnie : Tagbanan (Sénoufo)

Scolarisation : BAC+2 (Université)

Localité : Ogoudou

Profession : infirmier

**SECTION B : Connaissances et attitudes du personnel soignant**

B9 : Quelles sont les différents types de plaie que vous connaissez ?

A9 : Bon. Il y a les plaies traumatiques qui sont causées par les armes blanches (machettes, couteaux, pointes). Il y a des plaies simples qui sont dues à des furoncles ; le furoncle se forme puis se casse pour devenir une plaie. Nous avons les plaies qui sont communément provoquées par les mycobacterium ulcerans qui aboutissent à l’ulcère de Buruli.

B10 : Recevez-vous en consultation des patients porteurs de plaies ?

A10 : Bon on en reçoit. Mais ce n’est pas trop fréquent dans ces temps-là.

B11 : Si Oui, combien en moyenne par jours ? Mois ?

A11 : Il faut dire que ces temps-ci la fréquentation du centre n’est pas trop élevée. Cela est peut-être dû à la saison pluvieuse. On peut avoir au moins vingt à trente cas de plaies. Nous sommes deux infirmiers et moi je peux avoir vingt à trente. Combien lui il reçoit de patients, je ne sais pas.

B11 : Et pendant les saisons sèches ?

A11 : pendant les saisons sèches, le nombre augmente parce que beaucoup rentrent dans les basfonds pour les activités de riz et qui dit riz dit machette. Quand ils se blessent beaucoup, on a les plaies traumatiques. On peut avoir jusqu’à soixante patients.

B12 : Quelles sont les étiologies/causes des plaies que vous recevez ?

En dehors des plaies traumatiques dues aux armes blanches ?

A12 : Je vous ai parlé des furoncles. Il y a les accidentés de la voie publique qu’on reçoit souvent ; les plaies chirurgicales.

B13 : Quelles sont les différentes étapes par lesquelles passe une plaie avant de guérir selon vous ?

A13 : Bon si je comprends bien, tu demandes comment on procède aux soins de la plaie jusqu’à ce qu’elle guérisse totalement ? On le reçoit d’abord dans le registre pour l’identifier, pour qu’on sache que ce monsieur est arrivé effectivement au niveau de l’hôpital. Si nous avons les produits nécessaires pour faire le pansement, on le lui propose. S’il peut les acheter, ils les achètent et on le fait entrer dans la salle de pansement. Le pansement consiste à quoi ?

On fait un nettoyage de la plaie de l’intérieur vers l’extérieur avec les antiseptiques appropriés. Et puis si c’est une plaie qui n’est pas aussi grave, on utilise le sparadrap pour fermer ou bien une bande pour fermer. Et le pansement est fonction de l’état de la plaie. Ça peut-être une plaie grave et ça peut-être une plaie simple. Si c’est une plaie qui est grave, on fait le pansement de façon…. C'est-à-dire une plaie qui est grave, une plaie qui est suppurée. Quand elle est suppurée, on fait le pansement quotidiennement, au jour le jour jusqu’à ce que la suppuration puisse tarir. Une fois que la suppuration est éliminée, on lui fait un pansement chaque deux jours. Et après chaque trois jours jusqu’à ce que la plaie finisse totalement.

B13 : Donc si je comprends bien, est-ce que je peux dire que les étapes se résument en deux : il y a le nettoyage et le pansement. Et le nettoyage ou le pansement dépendent de l’état de la plaie.

B14 : Quels produits utilisez-vous couramment pour chaque étape ? Si nous prenons le nettoyage ?

A14 : C’est fonction de l’état de la plaie. On prend d’abord un cas de plaie simple. On peut utiliser le savon, le simple savon et puis on nettoie à côté. Une fois bien nettoyée, on ferme. Et le pansement peut se faire tous les jours. A défaut du savon, on peut utiliser le dakin simple. S’il n y a pas de dakin, on peut utiliser….là ce n’est pas forcément qu’on utilise le dakin dans les plaies simples hein ! Il faut que je le signale d’abord. Dans ces cas de plaies simples, on utilise la bétadine ; on fait le pansement avec la bétadine jusqu’à ce que ça finisse. Pour le cas d’une plaie suppurée, il faut chercher à faire tarir la suppuration. Il faut assécher ‘l’eau qui coule là’ ; on utilise dans ce cas du dakin.

B14 : Et pour le pansement ?

A14 : est-ce que le nettoyage est différent du pansement ?

B14 : moi je ne sais pas.

A14 : Nooon ! (rires). Une fois que vous avez nettoyé la plaie c'est-à-dire que le nettoyage se fait de l’intérieur vers l’extérieur. Vous enlevez les déchets dans la plaie. Une fois les déchets enlevés, si c’est une plaie suppurée, vous prenez une compresse avec du dakin que vous appliquez sur la plaie et vous attachez.

B15 : Quels sont les produits de pansements à votre disposition ?

A15 : Présentement, je n’ai que la bétadine. J’ai la bétadine, des compresses et les bandes. J’ai du sparadrap. Ça c’est pour les cas de plaies simples et souvent aussi traumatiques. Mais dans les cas de plaies suppurées, on prescrit le dakin parce qu’on ne l’a pas à la PSP (Pharmacie de la santé publique).

B17 : Quels sont selon vous les critères de changement des pansements ?

A17-A19 : Une plaie simple peut être due à un objet contondant c'est-à-dire un morceau de bois t’a tapé et ça va se gonfler et finalement s’ouvrir. Cela peut être dû à un objet pointu ou tranchant, ça te coupe et la personne vient à l’hôpital avec cette plaie. Généralement, cette plaie n’est pas profonde. C’est une plaie qui peut mesurer deux à trois centimètres de diamètre et un centimètre de profondeur. Je considère cela comme une plaie simple. On utilise donc des produits simples. On peut prendre le dakin pour le pansement.

B18 : Comment évaluez-vous l’évolution d’une plaie que vous prenez en charge ?

A18 : Quand vus soignez la plaie, vous regardez très bien, vous appréciez. Il y a une cicatrisation qui commence de l’extérieur vers l’intérieur. On sait que cela évolue favorablement et on encourage la personne à venir faire les pansements. Mais si vous avez utilisé un produit qui ne convient pas à votre malade, vous verrez que la plaie va devenir plus profonde, noire et cela va suppurer et va s’agrandir. Nous informons le patient et nous changeons automatiquement les produits que nous utilisons pour faire le pansement.

B20 : Prescrivez-vous des antibiotiques aux malades dans le cadre de la prise en charge de leurs plaies ?

A20 : Cela est possible et on le prescrit quelques fois. Mais dans les cas de plaies simples, on ne le prescrit pas. Dans le cas d’une plaie infectée et suppurée, cela est une obligation.

B21 : Si oui, quels antibiotiques prescrivez-vous ? Dans les cas de plaies suppurées,

A21 : Cela dépend du praticien. On prescrit des antibiotiques selon sa convenance et surtout selon la possibilité financière du client qui est en face de toi. Les moins chers que l’on peut utiliser sont l’amoxicilline, le cotrimoxazole, l’érythromycine.

B22 : Prescrivez-vous des antalgiques aux malades dans le cadre de la prise en charge de leurs plaies ?

A22 : je ne vois pas la nécessité de prescrire un antalgique. Je l’ai prescrit dans le cas du traitement d’une plaie d’une personne qui vient malheureusement de décéder. Et pour lui, c’était plutôt un sort qu’on lui aurait jeté. Si jamais la photo, j’allais vous la présenter (photo effacée). Chaque fois qu’on faisait le pansement au malade, il saignait, il se plaignait toujours de douleur. J’étais donc obligé sincèrement de lui prescrire des antalgiques.

B23 : Si oui, quels antalgiques prescrivez-vous ?

A23 : Tramadol en comprimé effervescent. Même dans les cas de plaies traumatiques, qui dit traumatisme dit douleur ; il faut obligatoirement prescrire des anti-inflammatoires. Le ‘Diclofénac’ est encore mieux parce que moins cher.

B 24-25 : Est-ce que vous référez les malades porteurs de plaies vers d’autres hôpitaux ? Vers quels hôpitaux ?

A24-25 : Cela arrive souvent. Vous pouvez recevoir un patient une ou deux fois, lorsque vous faites un traitement et qu’il n y a pas d’amélioration, à notre niveau, nous n’avons pas tous les appareils nécessaires pour les examens. On réfère donc le malade au CHR (Centre Hospitalier régional) de Divo pour une meilleure prise en charge.

B24-25 : Seulement au CHR ?

A24-25 : Ici à Ogoudou, monsieur Koné réfère au CHR de Divo.

B 26 : Est-ce que les hôpitaux de référence vous font une contre-référence ?

A26 : Moi j’ai les bulletins de référence mais on ne m’envoie jamais de contre-référence. Voulez-vous voir un bulletin de référence ?

B 27 : Souhaitez-vous être formé sur les soins des plaies ?

A27 : Il faut dire qu’on ne finit jamais d’apprendre. Je ne peux pas dire que je maitrise.

**SECTION C : Renforcement de capacité des agents de santé**

B29 : Si non souhaiteriez-vous être formé sur la prise en charge des plaies ?

Oui non

Si oui sur quels aspects ou quelles thématiques ?

A29 : je serai très ravi. Peut-être existe-t-il encore de nouvelles techniques de prise en charge de la plaie.

B29 : Peut-être que vous en connaissez ?

A29 : peut-être que je n’en connais pas non plus. Je sais qu’à l’école, nous avons appris les pansements secs, les pansements humides mais ce sont des choses qu’on rencontre très rarement. Tout à l’heure, vous êtes venus avec le médecin, je l’ai vu avec un bandage ; il est en train de faire un pansement humide. Ce n’est pas ça ?

B28 : Avez-vous jamais reçu une formation sur la prise en charge des plaies ?

A28 : Non. On a fait cela à l’école d’infirmerie. A part ça, on n’a jamais eu de formation. Donc voici les bulletins de référence mais quand on les remplit, il n y a pas de contre-référence.

B28 : Donc vous n’avez pas reçu de formation spécifiquement sur la prise en charge des plaies ?

A28 : On a reçu une formation sur l’ulcère de Buruli, la prise en charge de l’ulcère de Buruli parce que là, il y a des cas. Généralement dans les cas d’ulcère de Buruli, cela commence par des petits signes avant d’aboutir à l’ulcération. Tant qu’on n’a pas l’ulcération, on ne peut pas dire que c’est ulcération. Sinon, nous avons aussi été formés à la prise en charge d’un ulcère de Buruli.

B28 : Et cette formation avait duré combien de jours ?

A28 : est-ce que cela ne vaut pas une semaine ? Cela vaut une semaine.

B28 : Où ?

A28 : A Divo

B28 : Thème abordé

A28 : Prise en charge de l’ulcère de Buruli.

B28 : Comment avez-vous apprécié cette formation ?

A28 : C’était bien parce que cela nous a permis de connaitre vraiment beaucoup de choses sur l’ulcère de Buruli.

# **GUIDE D’ENTRETIEN PERSONNEL DE SANTE (COMMUNAUTE)**

Questionnaire No : ……………… Date de l’interview : ……/………/………

**SECTION A : INFORMATION SUR LE PERSONNEL DE SANTE**

1. Centre de santé :
2. Nom :

**SECTION B : Types de pratiques observées chez les patients et nature des questions posées par les patients**

B11 : Vous recevez des porteurs de plaies. A quel stade viennent-ils à l’hôpital ?

A11 : Cela dépend du malade. Il y a des gens qui n’aiment pas aller à l’hôpital. Ils ont la plaie, surtout les enfants, ils vont la cacher parce qu’ils craignent les antibiotiques injectables à l’hôpital. C’est lors que cela commence à se décomposer, ça sent et la personne pleure. Sa mère ou son père vient demander ce qu’il a et découvrir la jambe de son enfant. C’est déjà ‘gaté’. C’est là qu’on amène l’enfant.

B11 : Et les grandes personnes,

A12 : cela arrive aussi à certaines grandes personnes et cela dépend des moyens financiers. Ils disent qu’ils n’ont pas de l’argent ou bien ils ont honte que certains de leurs camarades sachent qu’ils ont une plaie. Donc ils préfèrent se cacher. Quand je dis ‘cacher les plaies’ ils se font soigner à l’indigénat. Ils utilisent généralement l’eau chaude. Après l’eau chaude, ils appliquent une feuille pour fermer. La chaleur décompose et quand cela devient grave, ils s’emmènent à l’hôpital.

B13 : Si oui, de quel traitement à domicile s’agit-il ? Vous donnent t ils le nom des feuilles,

A13 : Un traitement à l’indigénat. Sincèrement, moi je vois la feuille mais je ne connais pas le nom.

B15 : Dans l'affirmative, quelles sont les pratiques positives ou négatives les plus courantes en matière de soins des plaies, que ce soit au domicile ou ailleurs ?

A15 : Bon si ces feuilles que les patients utilisent à la maison étaient favorables pour la cicatrisation de la plaie, ils ne viendraient pas à l’hôpital. Cela est négatif.

B15 : Et pour l’eau chaude ?

A15 : l’eau chaude est tout juste pour….Il n’y a pas de savon dans l’eau chaude. Donc c’est tout juste pour un soulagement pendant quelques minutes. Ça n’a aucun effet sur la plaie.

B16-17 : Les malades ont-ils l’habitude de nettoyer leurs plaies ? Si Oui, qu'utilisent-ils ?

A16-17 : Certains utilisent l’eau chaude. Certains ont des canaris. Quand ils font leurs canaris, ils mettent le pied là-dessus selon la localisation. Soit la personne s’assoit, soit elle se couche, soit la personne met le membre ; l’essentiel serait que le canari qui a été bouilli là, la chaleur puisse ébouillanter la plaie et que cela permet à la plaie de vite finir.

B16 : Donc tout le monde ne nettoie pas nécessairement la plaie ?

A16 : j’ai même vu ici un patient qui avait une plaie. Bon la plaie était vraiment large, très large. J’ai commencé à faire les pansements, jusqu’à ce qu’il me dise « monsieur le docteur j’ai compris, tu as fait ce que tu pouvais faire mais il y a un monsieur qui peut me soigner à la maison » j’ai demandé si je peux aller assister et il m’a répondu oui. Excusez-moi hein ! Mais mes deux mains là ; c’est-à-dire que tu fermes sur la plaie mais cela ne peut pas. Mais quand je suis allé assister au pansement du monsieur, cela fait vomir. Il n’utilise rien mais sa langue. Il n’utilise sa langue pour soigner la plaie de l’enfant.

B17 : Comment le fait-il ? Cela m’intéresse.

A17 : (rires). Il lèche la palie comme ça….Et puis il avale. Et je t’assure qu’au bout de trois semaines, la plaie est finie. Il ne fait que lécher la plaie avec la langue. Je me suis dit par la suite que cela est de la sorcellerie pure. Cela n’est pas concevable qu’un être humain lèche la plaie de son semblable, allez savoir quelque chose (rires).

B18 : Les recouvrent-elles ou les bandent-elles ? Si Oui avec quoi ?

A18 : ça, ce sont les morceaux de pagne qu’ils utilisent, qu’ils attachent sous forme de bande.

B20 ; 21 ; 22 ; 23 : Est-ce que les malades posent des actes dangereux pour la santé ? Si oui quelles sont ces pratiques dangereuses ? Prennent-ils des produits / médicaments dangereux pour la santé ?

A20 ; 21 ; 22 ; 23 : Pourquoi pas ? Cela est possible. Quand ils ont leurs plaies à la maison, au lieu de venir nous voir directement, nous avons des petits concurrents dans le quartier qu’on appelle ‘les cliniques privées’. Ils vont les voir et demandent à faire une piqûre pour que la plaie finisse. On va certes te piquer mais si tu n’as pas soigné la plaie, va-t-elle guérir ? Et il lui vient en tête de faire soit la piqûre du tétanos, soit tu me fais la pénicilline pour que la plaie finisse, soit tu me fais le ‘retarpen’etc. Ils utilisent souvent le ‘toupaye’, l’amoxilline là, ils appellent cela ‘koungolili’ c'est-à-dire ‘tête rouge’. Quand ils finissent de faire leur pansement à l’eau chaude là, ils enlèvent la poudre là et ils appliquent là-dessus.

B21 : cela est-il dangereux pour la plaie ?

A21 Oui cela est dangereux. On n’utilise pas un antibiotique comme ça. Et ils ne sont pas encore des techniciens de santé. Le médicament qu’ils utilisent peut être périmé, la mauvaise conservation, quelle est la quantité qu’ils peuvent utiliser pour que leur plaie guérisse. Donc cela est mauvais pour l’utilisation.

B24-25 : Est-ce que les malades ont des interdits / restrictions alimentaires quand ils ont des plaies ? Si oui, quels sont ces interdits / restrictions alimentaires ?

A24-25 : Pour la guérison de leur plaie ? Tout à l’heure, je parlais du monsieur qui léchait les plaies là. On lui a interdit de manger beaucoup de choses. Tout ce qui est viande, poisson, que sais-je encore.

B26 : Quelles sont les pratiques des malades pour le soin des cicatrices ?

A26 : Bon ! Pratiquement je peux dire non. Mais la dernière fois, nous avons reçu la visite d’un médecin qui venait du Bénin ou du Togo. Il m’a demandé de faire venir les malades pour qu’il puisse voir les cicatrices. Il m’a conseillé après la cicatrisation d’une plaie d’utiliser du beurre de karité sur la cicatrice. Une fois appliqué, la partie cicatrisée devient molle et cela facilite un peu les mouvements. Donc pour ceux qui ne le savent pas, je ne peux pas confirmer qu’ils l’utilisent vraiment.

B27 : Quelles sont les pratiques des malades pour la prise en charge de la douleur ? Chez eux avant de venir à l’hôpital.

A27 : (rires). Bon ! Sincèrement, je n’ai aucune idée.

B28 : Quelles sont les pratiques des malades pour la prévention des invalidités ?

A28 : Oui. Généralement, c’est dans le cadre de l’ulcère de Buruli. Quand vous faites le pansement, il faut toujours faire les mouvements de flexion et extension si c’est au niveau du membre supérieur de sorte qu’après le traitement si la plaie est cicatrisée, que le membre atteint puisse être mobile.

Ceux qui viennent à l’hôpital, voilà ce qu’on leur dit de faire. Mais si tu ne viens pas te renseigner, tu vas rester évidemment dans l’ignorance.

B 29 : Est-ce qu’il y a des conceptions / idées fausses de la plaie et sa guérison chez les patients qui influencent leurs pratiques ou leurs recherches de traitement ? (ex : l’idée qu’il faut sécher la surface de la plaie). Lesquels ?

A29 : Si. Précisément dans les cas de l’ulcère de l’ulcère de Buruli. Je me rends compte qu’à mon ancien poste, on disait de ne pas aller à l’hôpital quand tu as cette plaie. Que lorsque tu vas à l’hôpital, on va te faire des piqûres et que la plaie ne finit pas. C’est typiquement le médicament africain qu’il faut faire. Et il y a des gens qui étaient des spécialistes dans ce genre de traitement. Ils se promènent de cours en cours pour chercher les malades. Mais ils ne te disent pas pourquoi ne pas venir à l’hôpital. Peut-être la seule raison est que la plaie ne finit pas vite. Pour l’ulcère de Buruli.

B30 : Quels types de question les malades vous posent-ils sur leurs blessures (plaie) ?

A30 : Les malades, lorsqu’ils viennent n’ont qu’une seule idée dans la tête, guérir.

Quand il guérit, s’il t a dit merci, Dieu merci. Une fois guéri, tu vas le voir dans la ville ; vous n’étiez pas là tout à l’heure, heureusement que j’ai pris le contact des patients que j’ai soigné. J’ai donc demandé à un de venir parce qu’il y a des ‘patrons ‘ qui viennent de « chez les blancs » qui veulent vous voir. Mais pour qu’ils viennent, cela était dur. J’ai dû prendre ma moto pour le chercher. Quand ils te voient dans la ville, ils disent « docteur bonjour. Merci » et c’est tout.

B31 : Est-ce qu’ils ont des préoccupations particulières concernant leurs plaies ou leurs traitements ?

A31 : Aucune question. Ils ne veulent rien savoir.

B32 : Les malades suivent-ils généralement les conseils que vous leur donnez ou suivent-ils le traitement recommandé ?

A32 : je pense qu’ils suivent les conseils.

B : Merci.
